# Canine olfactory detection of SARS-CoV-2-infected humans—a systematic review

**DOI:** 10.1016/j.annepidem.2023.05.002

**Published:** 2023-09

**Authors:** Sebastian Meller, Charles Caraguel, Friederike Twele, Marios Charalambous, Clara Schoneberg, Anne-Lise Chaber, Loïc Desquilbet, Dominique Grandjean, Fernando O. Mardones, Lothar Kreienbrock, Stéphane de la Rocque, Holger A. Volk

**Affiliations:** a Department of Small Animal Medicine & Surgery, University of Veterinary Medicine Hannover, Hannover, Germany; b School of Animal and Veterinary Sciences, The University of Adelaide, Adelaide, South Australia, Australia; c OIE Diagnostic Test Validation Science in the Asia-Pacific Region, The University of Melbourne, Melbourne, Victoria, Australia; d Department of Biometry, Epidemiology and Information Processing, WHO Collaborating Centre for Research and Training for Health in the Human-Animal-Environment Interface, University of Veterinary Medicine Hannover, Hannover, Germany; e École Nationale Vétérinaire d’Alfort, IMRB, Université Paris-Est, Maisons-Alfort, France; f École Nationale Vétérinaire d’Alfort, Université Paris-Est, Maisons-Alfort, France; g Escuela de Medicina Veterinaria, Facultad de Agronomía e Ingeniería Forestal, Facultad de Ciencias Biológicas y Facultad de Medicina, Pontificia Universidad Católica de Chile, Santiago de Chile, Chile; h Human Animal Interface, WHO Health Emergencies Programme, Geneva, Switzerland; i Center for Systems Neuroscience, Hannover, Germany

**Keywords:** SARS-CoV-2, COVID-19, Dog, Canine scent detection, QUADAS-2, Diagnostic test evaluation

## Abstract

**Purpose:**

To complement conventional testing methods for severe acute respiratory syndrome coronavirus type 2 infections, dogs’ olfactory capability for true real-time detection has been investigated worldwide. Diseases produce specific scents in affected individuals via volatile organic compounds. This systematic review evaluates the current evidence for canine olfaction as a reliable coronavirus disease 2019 screening tool.

**Methods:**

Two independent study quality assessment tools were used: the QUADAS-2 tool for the evaluation of laboratory tests’ diagnostic accuracy, designed for systematic reviews, and a general evaluation tool for canine detection studies, adapted to medical detection. Various study design, sample, dog, and olfactory training features were considered as potential confounding factors.

**Results:**

Twenty-seven studies from 15 countries were evaluated. Respectively, four and six studies had a low risk of bias and high quality: the four QUADAS-2 nonbiased studies resulted in ranges of 81%–97% sensitivity and 91%–100% specificity. The six high-quality studies, according to the general evaluation system, revealed ranges of 82%–97% sensitivity and 83%–100% specificity. The other studies contained high bias risks and applicability and/or quality concerns.

**Conclusions:**

Standardization and certification procedures as used for canine explosives detection are needed for medical detection dogs for the optimal and structured usage of their undoubtful potential.

## Highlights

• Dogs are able to detect SARS-CoV-2 infections via smell.
• Twenty-seven studies were evaluated by a semiquantitative (QUADAS-2) and a quantitative tool.
• Quality features included design, sample choice and properties, dogs, and training.
• Nonbiased studies showed >80% sensitivity and >90% specificity.
• Standardization of training procedures and certification of dogs is necessary.

## Introduction

The coronavirus disease 2019 (COVID-19) is a zoonotic infectious disease caused by the severe acute respiratory syndrome coronavirus type 2 (SARS-CoV-2), which was first detected in humans in China in late 2019 [1]. Within a few months, the disease spread rapidly due to unrestricted global mobility, becoming a worldwide pandemic. In addition to numerous fatal courses of the acute infection, it has also resulted in numerous recovered individuals suffering from long-term post-COVID-19 syndrome. The pandemic led to a major shift in the social and psychological [2], [3], [4], cultural [5], [6], economic [7], and political [8] landscapes, of which the full impact is not yet known. In the fight against the pandemic, counterstrategies were developed, including nonpharmaceutical physical and behavioral measures (e.g., hygiene measures, social distancing, protective masks, quarantine obligations, and lockdowns), diagnostic methods (e.g., real-time reverse-transcription polymerase chain reaction [RT-qPCR] and immunoassays for antigen or antibody detection), antiviral medication, and vaccines [9], [10]. However, the dynamics of the disease and the recent emergence of new variants of the virus emphasize that the individual measures and their combination need to be constantly adjusted as the pandemic develops [11]. With the intention of supplementing the SARS-CoV-2 testing repertoire, especially in its turnover, fitness-for-purpose, and affordability, the idea of using dogs’ sense of smell as a diagnostic screening tool was introduced in early 2020. Since then, more than 70 groups in 60 countries performed research on how dogs could be deployed to test people infected with SARS-CoV-2 [12].

Across human history, dogs' sensitive olfaction has been of great service primarily for hunting and guarding and later also for search and detection. Canids evolved with a very developed sense of smell, which was estimated to be 10,000–100,000 times more sensitive than in humans, with a lower limit of detection in the parts per trillion range [13]. Since the SARS-CoV-2 pandemic, many canine medical scent detection research groups have emerged. However, the potential of canine medical scent detection has already been highlighted for the detection of epileptic seizures [14], [15], hypoglycemia in patients with diabetes mellitus [16], cancer [17], bacteria (*Escherichia coli*, *Enterococcus*, *Klebsiella*, and *Staphylococcus aureus* in urine [18], as well as *Clostridium difficile* in stool [19]), malaria [20], and viral infections (e.g., bovine viral diarrhea virus in cell cultures [21]). More recently, this capacity has been tested for SARS-CoV-2, with samples of various body fluids [12]. The current general assumption is that metabolic alterations caused by infection and/or disease release a changed volatile organic compound (VOC) profile of the affected organism [22]. Those VOCs can be perceived and discriminated by dogs. The potential use of dogs' sense of smell for detection purposes in the medical field with attention to canine olfactory anatomy and physiology is reviewed elsewhere [12].

Considering the detection speed of only a few seconds, detection dogs have the potential for a fast turnover with real-time results and a high throughput compared to more conventional test methods. Those features are particularly interesting for screening scenarios (ruling-out context), in which dogs could systematically screen larger crowds of people before further individual tests could be conducted in order to confirm the condition (ruling-in context). However, dogs require to be accurate and fit-for-purpose to represent a compelling alternative to other tests. We systematically reviewed the scientific literature to summarize the current evidence on the fitness-for-purpose of detection dogs to screen SARS-CoV-2. For this purpose, we used two independent evaluation systems: Quality Assessment of Diagnostic Accuracy Studies 2 (QUADAS-2), according to Whiting et al., for a general assessment of the risk of bias and applicability of diagnostic accuracy studies, created for systematic reviews [23], and an adapted quality scoring tool according to Johnen et al. for a more specific evaluation of canine scent detection work [24], with an additional focus on medical scent detection. These two frameworks for assessment were chosen in order to analyze the dog like one would do for a laboratory-based diagnostic test (the semiquantitative QUADAS-2 tool for systematic reviews [23]) and to assess canines’ performance using a reporting framework for scent detection work (an adapted scoring tool by Johnen et al. [24]). Although score systems, as presented by Johnen et al. [24], are not considered to be adequate for systematic reviews [25], it was considered important to also assess here the operational and behavioral components of canines for medical scent detection and not only use a systematic review reporting system developed for laboratory diagnostics. It is further to note that both frameworks were applied separately and independently in the current study. Thus, both independent systems provide different perspectives on the review question and offer a complement and broader perspective. This approach should provide an idea of to which extent dogs perform well under realistic or “strict” conditions either as a diagnostic tool and/or as a detection dog. Elements such as patient selection, training methods, samples used, study design, canine breeds and characteristics, risks of bias, and statistical analysis methods used in the studies were assessed. In addition to high sensitivities and specificities in scent detection, further skills and characteristics of the dogs are essential. This is particularly true for large real-time population screening scenarios for rapidly spreading diseases such as COVID-19 (e.g., at airports and major events) as opposed to detection work in controlled laboratory conditions. Similar to explosives detection, the medical detection dogs will be exposed to various constantly changing environmental factors and deal with large screening populations, which requires efficient cognitive and behavioral performance, including high levels of training, motivation, obedience, team spirit, and health [26], [27]. Regarding medical detection, these aspects require further research and standardization procedures in order to effectively use medical detection dogs in screening scenarios following a procedure similar to canine explosives detection [28]. The review may form the basis for critically evaluating the medical usage of canine olfactory detection as a fast antiepidemic or antipandemic countermeasure. This may be vital in a world where epidemic diseases pose an ever-increasing threat [29].

## Material and methods

### Search strategy

The literature search aimed at sourcing all scientific studies evaluating the accuracy of detector dogs to screen people directly or through derived biological samples for SARS-CoV-2 infection. The Population, Intervention, Control, Outcome (PICO) framework was used to specify the review question. The following inclusion criteria were considered for evaluation:

i) *Type of study and study population (P): accessible peer-reviewed and preprinted articles of original or pilot studies (database search) or unpublished articles provided by the respective research group (manual search), in English or translated, were included. The study population consisted of infected individuals or derived human biological samples from individuals with SARS-CoV-2 infection and/or associated disease.*
ii) *Index test (I): canines (Canis lupus familiaris) represent the index test (or intervention) of interest, which was evaluated relative to a reference standard testing procedure.*
iii) *Reference standard (C): samples and odors, which were included in the disease discrimination and classification procedure by the dogs, derived from humans or human biological material subjected to PCR testing. To date, PCR testing procedures are considered the reference standard for SARS-CoV-2 detection and were, therefore, considered as the control in this review.*
iv) *Outcome (O): accuracy of the canine binary classifiers was reported using conventional measures, such as diagnostic sensitivity (SEN), specificity (SPE), and accuracy (ACC).*

Studies that did not meet the inclusion criteria were excluded, as were duplicates and preprints that were subsequently published in a peer-reviewed journal and, therefore, were considered obsolete. For the literature search, electronic search engines were used on databases such as PubMed (www.ncbi.nlm.nih.gov/PubMed), Web of Science (www.webofscience.com), CAB Abstracts (www.cabdirect.org), and Google Scholar (www.scholar.google.com) performed by one author (SM) from the current study. The following search terms were used in all mentioned electronic databases: ("canine detection" OR "dog detection" OR "detection dog" OR "detection dogs" OR "sniffer dog" OR "sniffer dogs" OR "scent dog" OR "scent dogs" OR "trained dog" OR "trained dogs" OR "tracking dog" OR "tracking dogs") AND (SARS-CoV-2 OR COVID-19). Manual searching for relevant data and papers was also performed, for example, via screening of reference lists of published literature and via correspondence with co-authors and expert groups in the field. All items retrieved from the search engines and manual searches were recorded and entered into the screening process. The authors have followed the Preferred Reporting Items for Systematic Reviews and Meta-Analyses (PRISMA) guidelines for diagnostic test accuracy during the development of the systematic review (www.equator-network.org).

### Study selection

The screening process was limited to studies published since 2019. A two-stage process of screening was applied:

STAGE 1—studies of relevance to the systematic review question were identified, which fulfilled the inclusion criteria i) and ii) and reported original findings related to scent dog detection of COVID-19-associated smell. At this stage, only the titles and abstracts of the studies were screened. Duplicates have been excluded at this stage. Titles and abstracts in non-English languages have been verified by a person who speaks the respective language and/or by using translation software (*DeepL Translator* and *Google Translate).*
STAGE 2—studies providing details for evaluation of applied methodology and robustness of outcome measures related to the review question were identified. At this stage, inclusion criteria iii) and iv) were verified by full-text screening. Subsequently, a thorough study assessment for complete data extraction was carried out for included studies. Published preprints were excluded when respective work was subsequently published in a peer-reviewed journal.

### Assessment of quality of diagnostic accuracy studies—QUADAS-2

For the assessment of individual studies’ quality of evidence, the QUADAS-2 tool for systematic reviews was used [23]. Two authors (SM and MC) individually and independently assessed all the studies based on the QUADAS-2 criteria to the best of their knowledge, and any potential disagreements were solved via a consensus between those two authors. This tool has been created for the evaluation of diagnostic accuracy studies. It guides appraisal of the risk of bias in four domains (patient selection, index test, reference standard, and flow and timing) and appraisal of applicability in three domains (the previously mentioned except flow and timing). Standard signaling questions in each domain help to assess low, high, or unclear risk of bias or applicability concerns (please refer to Whiting et al. [23] for additional information). The tool allows to adapt those signaling questions by omitting them or adding new ones to match the systematic review question and developed rating guidelines. In the current assessment, three signaling questions were added in the patient selection domain: a) *Were multiple sources of samples used?* b) *Were symptomatic and asymptomatic stages of disease included?* c) *Were other diseases/pathogens in positive or negative samples included?* An additional question was also included in the index test domain: d) *Was* “*novelty*” *of samples for the diagnostic test evaluation guaranteed?* These questions aim at a high variability in sample quality. High variability is to be considered favorable for the olfactory generalization of the dogs. The higher the variability of the olfactory confounding factors, the more robust is the learning of the respective target odor, and an adequate olfactory generalization process can be induced [30]. This enables the filtering of this odor from a myriad of individual and environmental odors that are inevitable in a large-scale open-world screening scenario. Confrontation of the dogs with novel (i.e., unknown) samples after the training phase is crucial to ensure a successful generalization process and represents an important quality feature for the diagnostic test evaluation (DTE). On the contrary, two signaling questions were omitted: a) *If a threshold was used, was it prespecified?* (index test) (excluded as currently not applicable) and b) *Was there an appropriate interval between index test and reference standard?* (flow and timing) (excluded as currently no information on adequate intervals is available in the scientific literature, especially with regard to possible long-lasting metabolism-induced odor changes of an infected individual). The assessment was conducted for each DTE in the included studies. A DTE was defined as the final test after a training phase from which SEN and SPE were extractable. In this context, it was possible that the assessed studies contained only one or multiple DTEs.

### Quality scoring of canine scent detection work based on Johnen et al.

In order to implement a second, numerical, and specific scoring system, an assessment tool for study quality of canine scent detection work was used, which was adapted and tailored to the review question and, therefore, to the field of medical canine scent detection [24]. The following categories were used: a) number of dogs involved, b) availability of relevant information on training, c) inclusion of novel samples in DTE, d) randomization of sample presentation, e) blinding, f) presentation of results, and g) critical discussion of results. Those categories were further supplemented with the following aspects: a’) study design, b’) sample characteristics or variability, c’) sample presentation repetition, d’) equality of treatment or preparation of positive and negative samples, and e’) olfactory transfer ability between different sample or virus inactivation types. While the original scores refer to the general quality of canine detection studies, the added categories target canine medical detection more specifically. Accordingly, the maximum scores in the individual categories have also been partially tailored to adjust the overall weighting (see following sections). The rating was based on a 17-point scale with 14.5–17, 9.5–14, and ≤9 points considered as high, medium, and low qualities according to the review question, respectively. Similarly, as for the QUADAS-2 assessment, every DTE was also evaluated by the system of Johnen et al. [24]. However, for comparability reasons, only the DTEs with the highest quality and/or relevance per study were compared across all studies (see also section Quality assessment: Canine scent detection work scores based on Johnen et al.). Further details on the rationales of scoring in mentioned categories are provided in the following sections.

#### Study design of diagnostic test evaluation (DTE)

Studies were categorized into three types with increasing potential for risk of bias and decreasing clinically meaningful information, that is, i) randomized comparative study, ii) cross-sectional (cohort) study, and iii) case-controlled study [31], [32], [33]. Assessment of quality across publications was scored for i) and ii) with one and for iii) with zero points. i) and ii) were considered equal in this evaluation since the main difference between both is the higher clinical relevance of i), which does not address the risk of bias for diagnostic accuracy [33]. If a cross-sectional design was clearly performed, but a part of the samples had to be added artificially to be able to evaluate the performance of the dogs at all, for example, because of low prevalence, 0.5 points were assigned. If the study design was not clear, zero points were given (category a’).

In addition, “microdesign,” that is, status of randomization (category d) and blinding (category e), was assessed for all studies. One point was given if randomization of sample positions in the canine test setting was ensured or zero points if it was not or not reported. Blinding status was scored with two points if DTE was double-blinded (i.e., neither the dog handler nor any other personnel, who was present during the test, knew about the status of the samples presented), one point if DTE was single-blinded (i.e., the dog handler did not know about sample status, but at least one other person in the scope of the testing event, who could interact with the dog handler or dog, knew about the sample status), or zero points if no blinding was conducted or no proper information was provided about its status [24]. Randomization and double-blind status were considered ensured in cross-sectional studies by default because the reference status of the samples was unknown when running the index test.

In addition, quality was scored in terms of the presentation of the results (category f) and their discussion (category g). Half a point was assigned to studies which at least presented results in a way that usual diagnostic metrics (at least SEN and/or SPE) could be extracted or calculated from displayed results. If results were presented in an unclear way, no points were assigned. In addition, if results were discussed with a focus on the potential risk of bias and/or study limitations, corresponding studies were given one additional half point. Less weight was given to these aspects as they do not have a direct impact on the dogs' performance.

#### Index test—Choice of dogs

The evaluated index test is unconventional since it is represented by living beings with differing individual needs and characters, and anatomical and physiological features, contrasting standardized and factory-made test systems. Therefore, the number of dogs used in the studies was considered relevant for the study quality assessment (category a) in order to mitigate those confounding factors of individual animals and, thus, allow for a more rigorous evaluation. The number of dogs was recorded for each DTE, and its potential impact was assessed according to the scoring system from Johnen et al., with one point given for one dog, two points for 2–4 dogs, and three points for five and more dogs deployed [24], [34].

Furthermore, prior experience of dogs might impact trainability on COVID-19-associated smell, as well as do anatomical and physiological features [26], [27], [35], [36]. Therefore, used breeds and past experiences with scent detection work were additionally assessed across all studies. Attention was paid to whether normocephalic (mesocephalic and dolichocephalic) or brachycephalic breeds were used and whether prior experiences were present or not. However, no points were provided for those aspects, as they are highly influenced by training duration and intensity, training paradigms, and canines’ learning styles that varied across studies.

#### Training

To assess quality related to training, particular attention was paid to whether sufficient information was provided to potentially being able to replicate the procedure (category b). When training protocol, including the rewarding nature (food, toy, and so on), or training strategy were provided, one point was assigned to the respective study and zero points when information was missing or unclear [24]. References to other publications in which the method had been described before were also credited.

In addition, the chosen training protocol was assessed as scent dog training paradigms differ depending on the search context used and may impact performance [37]. Furthermore, the duration of training was assessed since temporal limitations are of crucial importance in a pandemic situation.

#### Samples

##### Sample characteristics

As an important purpose of the review is to determine whether dogs can be used as a diagnostic test, the focus was also directed toward the selection of test samples (category b’). The hypothesis was that greater versatility in sample characteristics could lead to higher generalizability and an adequate generalization process for the target scent in the screening context [30]. Therefore, scores were provided in the following three subcategories: i) one point was given when more than two sources or facilities were used for both positive and negative samples or when there was a collecting point (e.g., screening center) where people with different backgrounds could provide samples. In the case of cross-sectional studies, the high versatility of the samples was considered to be ensured. ii) One point was provided when deliberately asymptomatic SARS-CoV-2-infected patients were considered in addition to samples from symptomatic COVID-19 patients and vice versa. In the case of cross-sectional studies, this aspect was considered warranted unless deliberate inclusions or exclusions of certain symptomatic conditions were made. iii) Finally, one point was provided if, in addition to healthy individuals, negative samples from individuals suffering from other diseases with symptoms similar to COVID-19 or other symptoms were included. For cross-sectional studies, this point was provided by default unless inclusion or exclusion criteria of certain symptom stages were explicitly stated. In all three subcategories, the respective point was not provided unless the criteria were fulfilled or if the characteristics of the sampling and the samples were not clearly described.

##### Repeated use of samples

It is not elucidated which qualitative and temporal aspects of an olfactory stimulus lead to the imprinting of information and to which extent this olfactory information can be memorized [38]. It is important to exclude as best as possible that dogs recognize individuals rather than the actual infection status, which is the objective of olfactory generalization in the training process [30]. Therefore, quality was scored in two categories: the first one dealing with the use of new or unknown samples in DTEs after the training phase (category c) and the second one with repetition of samples (repeatedly screened by the same dog during evaluation; category c’). When dogs were presented with new samples for the DTE, the respective study obtained two points. When no information about novelty was provided, attention was paid to potential quantitative and temporal discrepancies in the general amount and processing of samples recruited versus samples used for training versus DTE. Zero points were given when novelty could not be guaranteed. In terms of sample repetition, one point was given if repetitions in the conduct of the DTEs could be excluded or, if not excluded, corrections for repetitions were conducted in respective outcomes. When no information about repetitions was provided, the focus was directed toward potential quantitative and temporal discrepancies in the general amount and processing of samples recruited versus samples used for training versus DTE. Zero points were given when the exclusion of repetitions could not be guaranteed. The samples’ novelty aspect between training and DTEs is awarded with more points than the exclusion of repetitions in DTEs because the novelty aspect is considered to have a more pronounced impact that can influence the performance of the dog.

##### Sample preparation

To consider possible fundamental differences in sample preparation, which could influence dogs’ performance, such as inactivation procedures, the focus was directed to whether positive and negative samples for DTEs were treated equally (one point). Zero points were provided if positive and negative samples were treated differently or if biological samples were tested against nonbiological samples (e.g., clean or unused swabs, gauzes, and masks) or if the information was too sparse (category d’). If studies assessed whether sample type or inactivation procedures of samples used in training impacted the recognition of other sample types or noninactivated samples (i.e., similar to real field samples) used during DTEs or, more generally, if olfactory transfer performance from one type of sample or inactivation method used in training to another type of sample or inactivation method used in the DTE was addressed, one additional point was given (category e’). The viral inactivation methods used for safety reasons were also assessed.

### Assessment of quality of outcome measures

The following result metrics were extracted from the publications: SEN, SPE, and ACC (number of true positive and negative canine indications/number of all indications). If those metrics were only partially reported, missing metrics were calculated from the provided data. In a first attempt, contingency tables were extracted per dog involved in each DTE, and the median, interquartile range, and overall range of the mentioned metrics were calculated or adopted among dogs for each DTE. If it was not possible to resolve the data by individual dogs (e.g., due to a single general contingency table reported or due to the definition of sample status by screening of more than one dog), overall metrics were adopted or calculated from the common contingency table per DTE. Calculations were conducted with the Prism 9 software (GraphPad, La Jolla, CA). Details of data extraction for each study are additionally reported in Supplementary Table 1.

## Results

### Search results

**Fig. 1:**
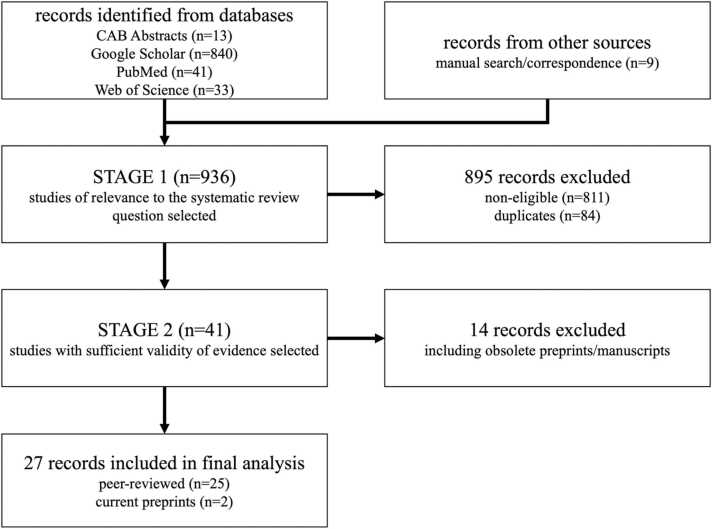
Flow diagram of study selection for the systematic review, as well as study inclusion and exclusion process for final analysis.

The literature search for corresponding original studies published between 2019 and 2022 yielded 13 records on CAB Abstracts, 840 records on Google Scholar, 41 records on PubMed, and 33 records on Web of Science. Nine records were considered for analysis from the manual search of reference lists and correspondence. In total, after excluding the non-eligible records, duplicates, and obsolete preprints or manuscripts that were later published in peer-reviewed journals, 27 reports with the respective inclusion criteria were found and entered in the final analysis. Those included two current preprints (Fig. 1). Studies are from 15 countries and differ in their designs. Ten DTEs of a total of 59 DTEs among the 27 studies were conducted as cross-sectional test evaluations [39], [40], [41], [42], [43], [44], [45]. Supplementary Table 1 provides a detailed overview of assessed features for each study and DTEs, while Supplementary Table 2 provides a general overview of DTE characteristics. Supplementary Table 3 provides an overview of the excluded studies at Stage 2.

### Quality assessment: QUADAS-2 for diagnostic accuracy studies

All included 27 studies were analyzed by the QUADAS-2 assessment of quality of diagnostic accuracy studies [23]. The analysis was conducted for every DTE run across all studies, resulting in overall 59 DTEs for assessment. The assessment of the risk of bias and applicability concerns in the differing domains is presented in Table 1. Overall, seven DTEs distributed over four studies from Hag-Ali et al. [39], Grandjean et al. [42], Kantele et al. [43], and ten Hagen et al. [45] had the least overall risk of bias and the best applicability. The studies by Wurtz et al. [41] and Maurer et al. [44] were not included despite a supposed low bias because only a little information was provided in the former study, and only one dog was used in the respective follow-up DTE of the latter study.

**Table 1:** QUADAS-2 assessment for quality and applicability of evidence in diagnostic accuracy studies [23]

Report*	Ref		Risk of bias				Applicability concerns		
			Domain I Patient selection	Domain II Index test	Domain III Reference standard	Domain IV Flow & timing	Domain I Patient selection	Domain II Index test	Domain III Reference standard
Jendrny et al. (2020)	[46]								
Grandjean et al. (2020)	[47]								
Eskandari et al. (2021)	[48]	−DTE 1							
		−DTE 2							
Grandjean et al. (2021)	[49]								
Essler et al. (2021)	[50]	−DTE 1							
		−DTE 2							
		−DTE 3							
		−DTE 4							
		−DTE 5							
		−DTE 6							
		−DTE 7							
		−DTE 8							
Hag-Ali et al. (2021)	[39]								
Mendel et al. (2021)	[51]	−DTE 1							
		−DTE 2							
		−DTE 3							
Angeletti et al. (2021)	[52]								
Sarkis et al. (2022)	[53]								
Vlachová et al. (2021) (*pre*)	[54]								
Jendrny et al. (2021)	[55]	−DTE 1							
		−DTE 2							
		−DTE 3							
		−DTE 4							
		−DTE 5							
Wurtz et al. (2021) (*pre*)^†^	[41]		()	()	()	()	()	()	()
Vesga et al. (2021)	[40]	−DTE 1							
		−DTE 2							
		−DTE 3							
		−DTE 4							
Maia et al. (2021)	[56]								
ten Hagen et al. (2021)	[57]	−DTE 1							
		−DTE 2							
		−DTE 3							
Grandjean et al. (2022)	[58]								
Grandjean et al. (2022)	[59]								
Devillier et al. (2022)	[60]	−DTE 1							
		−DTE 2							
Chaber et al. (2022)	[61]								
Guest et al. (2022)	[62]	−DTE 1^‡^							
		−DTE 2^‡^							
		−DTE 3							
Mancilla-Tapia et al. (2022)	[63]	−DTE 1							
		−DTE 2							
Maurer et al. (2022)	[44]	−DTE 1							
		−DTE 2^§^	()						
Kantele et al. (2022)	[43]	−DTE 1							
		−DTE 2^§^	()						
Grandjean et al. (2022)	[42]								
Twele et al. (2022)	[64]	−DTE 1							
		−DTE 2							
		−DTE 3							
ten Hagen et al. (2022)	[45]	−DTE 1^§^	()						
		−DTE 2^§^	()						
		−DTE 3^§^	()						
		−DTE 4^§^	()						
Demirbas et al. (2023)	[65]	−DTE 1							
		−DTE 2							
		−DTE 3							

DTE = diagnostic test evaluation; Pre = preprint; Ref = reference.

low risk or concerns;  high risk or concerns;  unclear risk or concerns.

*Reports sorted by publication date. ^†^Not enough information for appropriate assessment. ^‡^DTE described in appendix. ^§^DTE was conducted with cross-sectional patient selection and study design. However, additional samples had to be added to the study process due to low prevalence.

### Quality assessment: Canine scent detection work scores based on Johnen et al.

Table 2 provides scores for the assessment of study quality in canine scent detection work adapted from Johnen et al. [24]. Not all of the 59 originally analyzed DTEs (see Supplementary Tables 1 and 2) were considered for this evaluation since each study was assessed as one coherent entity. This appears reasonable for comparison between studies, as some reported DTEs were considered preliminary in anticipation of a main DTE per study (chaining of multiple successive DTEs, with the last DTE considered as the main outcome according to the study hypothesis, design, and presentation of results) [40], [43], [44], [62], [51], [65]. In other studies, procedures of multiple DTEs had rather similar or parallel features (multiple DTEs conducted as parallel study arms with each DTE considered as a main outcome according to the study hypothesis, design, and presentation of results) [45], [48], [55], [57], [60], [63], [64], that is, dogs were trained and tested in multiple independent DTE scenarios (e.g., with differing sample types). Other reports with multiple DTEs had intermediate characteristics [50]. Finally, the majority of publications consisted of only one training followed by one DTE [39], [41], [42], [47], [46], [52], [49], [56], [54], [61], [58], [59], [53] (see also Supplementary Table 2 for a general DTE overview).

**Table 2:** Modified scoring system for numerical assessment of study quality of canine scent detection work [24]

Report*	Ref	Study design [I]	No. of dogs (score) [II]	Relevant information on training given? [III]	Sample characteristics/variability in DTE [IV]	Use of novel samples in DTE? [V]	Sample presentation repetition in DTE? [VI]	Equal sample treatment/preparation in DTE? [VII]	Olfactory transfer between different sample/inactivation types addressed? [VIII]	Randomization in DTE? [IX]	Blinding in DTE [X]	Presentation of results [XI]	Critical discussion of results [XII]	Total/max. score	Total/max. score in %
Jendrny et al. (2020)	[46]	0	3	1	1	2	0	1	0	1	2	0.5	0.5	12/17	70.6
Grandjean et al. (2020)	[47]	0	3	1	1	2	0	1	0	1	1	0.5 (*suppl*)	0.5	11/17	64.7
Eskandari et al. (2021) (all DTEs)	[48]	0	2	0	2	0	1	1	0	0	1	0.5	0.5	8/17	47.1
Grandjean et al. (2021)	[49]	0	3	1	2	2	1	1	0	1	1	0	0.5	12.5/17	73.5
Essler et al. (2021) (sixth DTE)	[50]	0	3	1	1	2	0	1	0	0	1	0.5	0.5	10/17	58.8
Hag-Ali et al. (2021)	[39]	1	2	1	3	2	1	1	0	1	2	0.5	0.5	15/17	88.2
Mendel et al. (2021) (third DTE)	[51]	0	2	0	0	0	0	0	0	1	1	0.5	0.5	5/17	29.4
Angeletti et al. (2021)	[52]	0	2	1	1	0	0	1	0	1	0	0	0	6/17	35.3
Sarkis et al. (2022)	[53]	0	2	0	2	2	1	1	0	1	1	0.5	0.5	11/17	64.7
Vlachová et al. (2021) (*pre*)	[54]	0	2	0	0	0	0	1	0	1	1	0.5	0	5.5/17	32.4
Jendrny et al. (2021) (all DTEs)	[55]	0	3	1	3	2	0	1	1	1	2	0.5	0.5	15/17	88.2
Wurtz et al. (2021) (*pre*)	[41]	1	1	0	3	2	1	0	0	1	2	0.5	0	11.5/17	67.6
Vesga et al. (2021) (third DTE)	[40]	0	3	1	3	2	1	1	0	1	1	0.5	0.5	14/17	82.4
Maia et al. (2021)	[56]	0	2	0	2	0	1	1	0	1	2	0.5	0	9.5/17	55.9
ten Hagen et al. (2021) (first DTE)	[57]	0	3	1	2	2	0	1	1	1	2	0.5	0.5	14/17	82.4
Grandjean et al. (2022)	[58]	0	3	1	2	2	1	1	0	1	2	0.5	0.5	14/17	82.4
Grandjean et al. (2022)	[59]	0	2	0	1	0	0	1	0	1	1	0.5	0	6.5/17	38.2
Devillier et al. (2022) (second DTE)	[60]	0	2	1	2	2	1	1	1	1	1	0.5	0.5	13/17	76.5
Chaber et al. (2022)	[61]	0	3	1 (*suppl*)	1	2	1	1	0	1	2	0.5	0.5	13/17	76.5
Guest et al. (2022) (third DTE)	[62]	0	3	1	3	2	1	1	0	1	2	0.5	0.5	15/17	88.2
Mancilla-Tapia et al. (2022) (first DTE)	[63]	0	2	1	2	2	1	1	1	1	1	0.5	0.5	13/17	76.5
Maurer et al. (2022) (first DTE)	[44]	0	2	1	3	2	1	1	0	1	2	0.5	0.5	14/17	82.4
Kantele et al. (2022) (second DTE)	[43]	0.5	2	1	3	2	1	1	0	1	2	0.5	0.5	14.5/17	85.3
Grandjean et al. (2022)	[42]	1	3	1	3	2	1	1	0	1	2	0.5	0.5	16/17	94.1
Twele et al. (2022) (first DTE)	[64]	0	3	1	2	2	0	1	1	1	2	0.5	0.5	14/17	82.4
ten Hagen et al. (2022) (second to fourth DTE)	[45]	0.5	3	1	3	2	1	1^†^	1	1	2	0.5	0	16/17	94.1
Demirbas et al. (2023) (third DTE)	[65]	0	2	1	1	0	0	0	1	0	0	0.5	0.5	6/17	35.3

DTE = diagnostic test evaluation; Pre = preprint; Ref = reference; Suppl = supplementary material.

Studies with high quality (14.5–17 points [>85%]), medium quality (9.5–14 points [55%–85%]), and low quality (≤9 points [<55%]) in accordance with the review question.

I Diagnostic accuracy comparative study or cross-sectional study (1 point), cross-sectional study with addition of predefined samples, for example, because of low prevalence (0.5 points), case-controlled study or no information or unclear (0 points).

II ≥5 Dogs (3 points), 2–4 dogs (2 points), and one dog (1 point).

III Relevant training information available (1 point), and no or unclear training information available (0 points).

IV Multiple sample sources (+1 point), symptomatic and asymptomatic patients (+1 point), and patients tested negative with other disease or COVID-19 similar symptoms (+1 point), if conditions do not apply or no information (0 points for each).

V Novel samples for DTE used (2 points), known samples for DTE used or no information (0 points).

VI No sample repetitions in DTEs (1 point), and repetitions present in DTEs or no information (0 points).

VII Positive and negative samples in DTEs treated equally (1 point), samples not treated equally or biological samples tested against nonbiological samples, or no information (0 points) (†Authors showed before that sample inactivation [beta propiolactone] did not impact canine olfaction in terms of detecting noninactivated samples [55]).

VIII Olfactory transfer performance from one type of sample or inactivation method used in training to another type of sample or inactivation method used in the DTE addressed (1 point) and equal sample types or inactivation methods used in training and DTE (0 points).

IX Randomization of sample positions (1 point), and no randomization or no information (0 points).

X Double blinding of sample positions (2 points), single blinding (1 point), and no blinding or no information (0 points).

XI At least sensitivity and specificity available or extractable (0.5 points) and less information available or unclear (0 points).

XII Discussion about limitations and risk of bias (0.5 points), and no critical discussion available (0 points).

*Reports sorted by publication date.

In the case of a study with multiple successive DTEs, the final DTE was chosen for analysis. Vesga et al. finished their study with two similar DTEs. The penultimate DTE was chosen (third one) because there was a long training break for dogs (2.5 months) before the last DTE, so the third DTE was considered more comparable to the other studies [40]. A further exception is the study of Maurer et al., where the first DTE was chosen since the study question focused on the corresponding DTE, whereas the second DTE was defined only as a follow-up phase with only one dog being involved [44].

In the case of a study with the performance of multiple similar or parallel DTEs, quality scores were of equal value due to unchanged conditions in two studies [48], [55], so that all DTEs were considered. However, some qualitative differences between independent DTEs of the same study were present, and therefore, DTEs with the highest quality per study according to the review question were chosen for representative comparison in Table 2: first DTE of ten Hagen et al. [57], second DTE of Devillier et al. [60], first DTE of Mancilla-Tapia et al. [63], first DTE of Twele et al. [64], and DTE 2–4 of ten Hagen et al. [45]. For the DTEs from Essler et al., who conducted differing successive and parallel designs, the DTE with the most robust study design (sixth DTE) was chosen [50].

Six studies obtained the status of high quality [39], [42], [43], [45], [62], [55], whereas 15 were assigned a medium quality [40], [41], [44], [57], [60], [63], [64], [50], [47], [46], [49], [56], [61], [58], [53], and six were scored with low quality [51], [65], [48], [52], [54], [59] in accordance to the review question. Detailed scoring in the chosen 12 categories and total scoring are presented in Table 2. More details about DTE characteristics and scoring can be obtained from Supplementary Table 1.

### Comparison of different study characteristics

In order to provide a comparative overview of the working methods across all studies, determinant features of canine scent detection work have been extracted, including the selection of dogs, the training methods, and the sample material. In addition, those individual features were compared in the studies with the least risk of bias and the highest quality according to QUADAS-2 [23] and to the evaluation system of Johnen et al., respectively [24]. See Supplementary Table 1 for details.

#### Choice of canines

**Fig. 2:**
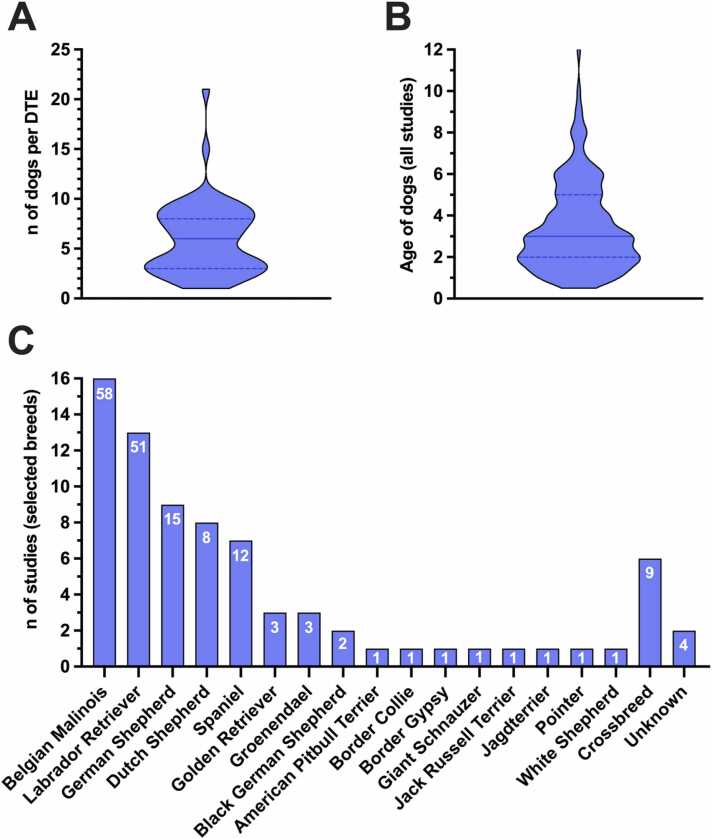
The violin plot in (*A*) shows the number of dogs involved in each diagnostic test evaluation (DTE; n = 59) from the evaluated 27 studies. The violin plot in (*B*) shows the age of involved dogs across all studies at the time of performing the DTEs, resulting in n = 158 involved dogs (partially repeated, see below). Age was not mentioned in some studies resulting in exclusion from (*B*). Lines in the violin plots represent the median (*solid lines*) and quartiles (*dashed lines*). The bars in (*C*) represent the number of studies in which the mentioned breeds were involved. The numbers inside the bars represent the overall number of involved dogs of respective breeds across all studies. Please note that some dogs have been repeatedly involved in follow-up studies from the same working groups, resulting in repeated appearance of individual dogs’ data in (*B*) and (*C*). If age was not mentioned, it was estimated based on previously conducted studies with the same dogs.

The median number of dogs involved in DTEs across all studies was 6 (range 1–21) (Fig. 2*A*). Overall estimated median of reported dog age was 3 years (range 0.5–12) at the time the DTEs were conducted, although some dogs might have been involved multiple times in follow-up studies from the same working group, and in some cases, age was estimated based on previous studies (Fig. 2*B*). All reported dog breeds belonged to normocephalic breeds. Belgian Malinois was involved in 16, Labrador Retriever in 13, German Shepherd in nine, Dutch Shepherd in eight, Spaniel in seven, each Golden Retriever and Groenendael in three, Black German Shepherd in two, and each American Pitbull Terrier, Border Collie, Border Gypsy, Giant Schnauzer, Jack Russell Terrier, Jagdterrier, Pointer, and White Shepherd in 1 of the 27 reviewed reports. Crossbreed dogs were involved in six of the studies, whereas breed was not mentioned (or could not be estimated based on previous studies) in two of the studies (Fig. 2*C*). Previous experience in scent detection work was present in all dogs in 8 of 27 studies, a varied background experience level in 11 of 27 studies, and the absence of previous experience in two studies. Dogs had COVID-19 detection experience from previous studies in 6 of the 27 studies. Information about previous experience levels was not provided in 6 of 27 studies. See also Supplementary Table 1.

##### QUADAS-2 assessment of studies with least risk of bias: Canines

In the four reports with the least risk of bias and best applicability to our review aim [39], [42], [43], [45] (Table 1), the commonly involved breeds were Belgian Malinois, German Shepherd, and Labrador Retriever. In detail, Hag-Ali et al. included one Belgian Malinois, one German Shepherd, and two Belgian Malinois/German Shepherd crossbreeds (n = 4, age not provided) [39], and ten Hagen et al. included three Belgian Malinois, two Labrador Retrievers, one German Shepherd, and two German Shepherd crossbreeds (n = 8, median age 4 years, range 2–10) [45]. Kantele et al. included three Labrador Retrievers and one White Shepherd (n = 4, median age 5.5 years, range 4–8) [43]. Previous experience level among dogs was present [39], [42], [43] or varied [45]. SARS-CoV-2 detection experience was present in the studies from Grandjean et al. [42] and ten Hagen et al. [45] based on previous studies. Grandjean et al. involved seven dogs in their study (six Belgian Malinois, one Groenendael, estimated median age of 4 years, range 3–6.5). Although this information was not provided in the actual report or supplementary material [42], age and breed could be estimated from previous publications from the same working group [49], [58].

##### Assessment of high-quality studies based on Johnen et al.: Canines

Among the six reports classified with high quality according to the adapted scoring system by Johnen et al. [24] (see also Quality assessment: Canine scent detection work scores based on Johnen et al. and Table 2), Belgian Malinois was involved in four studies [39], [42], [45], [55], Labrador Retriever was involved in four studies [43], [45], [62], [55], German Shepherd was involved in three studies [42], [45], [55], and each Dutch Shepherd [55], Cocker Spaniel [62], Golden Retriever [62], Groenendael [42], and White Shepherd [43] were involved in one study. Crossbreeds were involved in three studies [39], [45], [62]. The median number of dogs across the assessed DTEs (see Quality assessment: Canine scent detection work scores based on Johnen et al.) was 7.5 (range 4–10). The median age of all included dogs across the studies where age was reported or could be estimated was 4 years (range 1–10), although some dogs might have been involved multiple times in follow-up studies from the same working group. Previous experience was present in three studies [39], [42], [43], whereas experience level was varied in two studies [45], [55] and unknown in one study [62]. Previous SARS-CoV-2 detection experience was present in three studies [42], [45], [55].

#### Choice of training paradigms

**Fig. 3:**
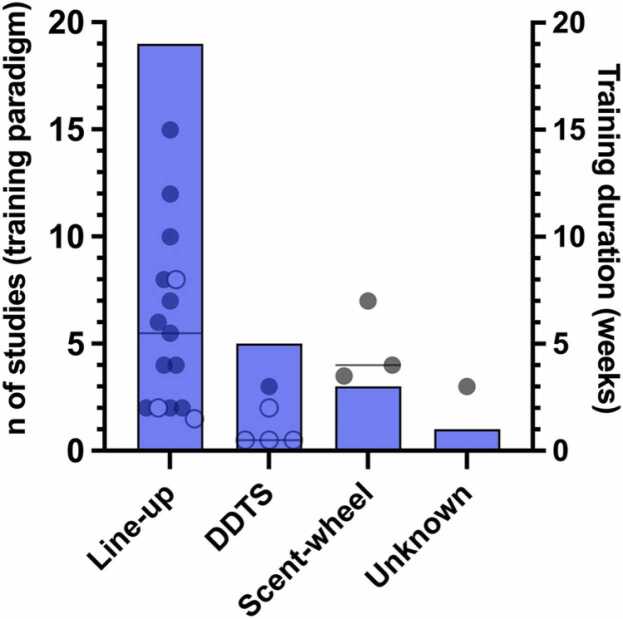
Bars show the number of studies in which the mentioned training paradigms were used (left *y*-axis). The scatter dot plot represents the approximate duration of training (weeks; right *y*-axis) per study and per training approach. Please note that the training duration is only an approximation due to partly unclear statements across the studies. The median of training duration is represented by *solid horizontal lines*. *Open circles* represent training duration in studies where dogs had previous COVID-19 detection experience. Some studies did not report training duration and were excluded from the graph (n = 4). Please note that one study used line-up and Detection Dog Training System (DDTS) approaches [45].

Overall, three different training paradigms were used in the studies. Line-up training was conducted in 19 of 27 reviewed studies, whereas training with Detection Dog Training System (DDTS) was conducted in five and scent-wheel training in three studies. One study did not report the sample presentation approach [41] (Fig. 3). One of the reviewed reports used line-up and DDTS in their training approach [45]. If reported, the reward for the detection of a positive sample was mainly immediate across the studies with a balanced use of food-reward and/or nonfood-reward approaches, such as toys or praise.

Assessment of the duration of training was complex due to partly missing information (e.g., only minimal duration or time periods provided) or due to possibly missing information about the time of habituation training before COVID-19 scent imprinting. If minimal duration was provided, the respective value was included in calculations, and if time periods were provided, the corresponding averaged time value was included. If multiple successive DTEs with the same dogs were performed in one study, the durations per DTE were added up. The approximate median time of overall training was 3.75 weeks (range 0.5–15): 5.5 weeks (range 1.5–15) for line-up, 0.5 weeks (range 0.5–3) for DDTS, and 4 weeks (range 3.5–7) for scent-wheel (Fig. 3). Four studies did not contain any information about training time. After the exclusion of six studies where preceding COVID-19 scent detection experience in dogs was reported or evident, the overall median approximate training time was 4 weeks (range 2–15): while the scent-wheel paradigm was not affected by the exclusion, the median training duration for the line-up paradigm increased to 5.75 weeks (range 2–15). After exclusion, only one study without prior COVID-19 detection experience remained in the DDTS paradigm group with approximately 3 weeks of training duration (2 weeks of habituation and 1 week of imprinting) [46]. See also Supplementary Table 1.

##### QUADAS-2 assessment of studies with least risk of bias: Training paradigms

For the four studies with DTEs of least risk of bias and best applicability (Table 1), Hag-Ali et al., Grandjean et al., and Kantele et al. used line-up [39], [42], [43], while ten Hagen et al. used DDTS and line-up scenarios for training purposes [45], respectively. Hag-Ali et al. trained dogs for approximately 5.5 weeks [39] and Grandjean et al. for 1–3 weeks [42], while ten Hagen et al. trained dogs 3 days at the DDTS and 1–2 weeks at the line-up [45]. Dogs with preceding COVID-19 detection experience participated in latter study. Kantele et al. did not report training duration but stated that dogs were considered prepared when success rates of >80% were achieved during training [43].

##### Assessment of high-quality studies based on Johnen et al.: Training paradigms

Among the six reports classified with high quality according to the adapted scoring system by Johnen et al. [24] (see also Quality assessment: Canine scent detection work scores based on Johnen et al. and Table 2), line-up training was used in five studies, while DDTS was used in two studies (both approaches were used in ten Hagen et al. [45]). Kantele et al. did not report any training duration [43]. Overall median approximate training duration across the remaining five studies was 2 weeks (range 2–7), taking into account the total training duration from previous DTEs for Guest et al. [62] and the only coherent training phase for all assessed DTEs in the studies from Jendrny et al. and ten Hagen et al. [45], [55]. Importantly, dogs with preceding COVID-19 detection experience participated in the latter two studies and in the study from Grandjean et al. [42]. In the two remaining studies, in which the dogs had no prior COVID-19 detection experience, the training duration was approximately 5.5 weeks [39] and 7 weeks [62], respectively.

#### Choice of sample types and inactivation method for DTE

During the DTE phases, the following sample types were used across all 27 studies: a) different types of sweat samples in 20 of reviewed studies (12 studies with axillary sweat, 4 studies with sweat from the crook of the arm or the wrist, 2 studies with corporal or torso sweat, 2 studies with sweat from the head or face area, and 2 studies with clothes), b) saliva samples in six, c) masks or breath samples in six, d) upper airway samples in four, e) urine samples in three, f) direct body sniffing in one, and g) other biological samples such as cell cultures in 1 of 27 reviewed studies. In one study, too little information about the sample type was provided [41]. While sampling of saliva, urine, upper airway samples, and direct sniffing of body odor generally takes seconds to minutes, the sample acquisition time of body contact-related samples, such as sweat (e.g., cotton pads and clothes) or breath (e.g., masks), varied across studies. Despite partly inaccurate information comparable to the reporting of training duration (see Choice of training paradigms), only a rough estimate of the acquisition time of those samples can be provided: axillary sweat sampling across 10 studies took approximately a median of 10 minutes (range 1–20) [42], [47], [49], [52], [56], [58], [59], [60], [61], [63], and 2 studies did not report sampling time of axillary sweat [39], [53]; corporal or torso sweat sampling was conducted in 2 studies and took 1 [63] or 20 minutes [54]; and the duration of sampling of sweat from the arm crooks, wrists, and head or face area took only seconds [43], [44], [45], [55], [64]. Clothes were worn for 12 hours (socks) [62] or 24 hours [48] before sampling them. The median acquisition duration of mask or breath samples was 107.5 minutes (range 3 minutes to 24 hours) [62], [51], [65], [48], [60], [54].

**Fig. 4:**
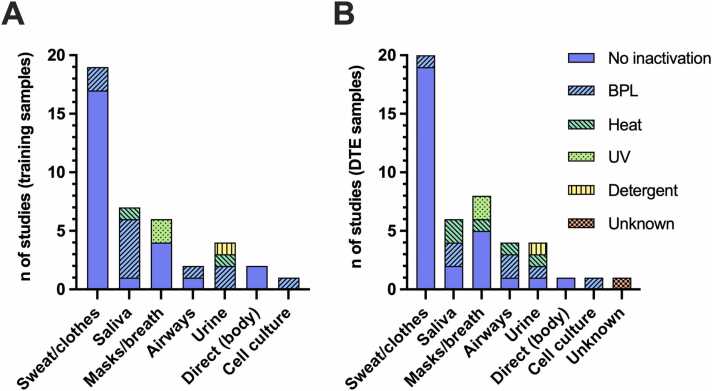
Bars represent the number of studies with used combinations of sample types (horizontal axis) and inactivation types (colors) for SARS-CoV-2 positive samples in at least one trial of the corresponding study. (*A*) shows the sample and inactivation combinations for training samples, while (*B*) represents combinations used in the diagnostic test evaluations (DTEs). Please note that individual studies might have used multiple combination options. BPL, beta propiolactone; UV, ultraviolet radiation.

The following inactivation methods were used for positive samples used in DTEs: a) no inactivation was used in 21, b) beta propiolactone (BPL) was used in three, c) heat treatment was used in three, d) ultraviolet light (UV) was used in two, and e) detergent inactivation (NP-40) was used in 1 of the 27 reviewed studies. Details about inactivation procedures were sparse in one study [41]. In 9 of the 27 reviewed studies (i.e., in 25 DTEs of overall 59 DTEs), either the type or the inactivation procedure of training samples differed from the samples used in DTE in at least one trial of respective study [40], [45], [65], [55], [57], [60], [63], [64], [50], which had no significant impact on canine detection performance. Figure 4 represents the used combinations of sample and inactivation types across all studies for both the training (Fig. 4A) and evaluation phases (Fig. 4B). See also Supplementary Tables 1 and 2 for more information.

##### QUADAS-2 assessment of studies with least risk of bias: Samples

During the DTEs, Hag-Ali et al. and Grandjean et al. used axillary sweat with unclear acquisition time [39] or 2 minutes of acquisition time [42], while ten Hagen et al. and Kantele et al. used sweat from the crook of the arm [45] or from the wrist and head or face area [43] obtained within seconds. In all four studies, the respective DTEs were performed on noninactivated samples (Table 1).

##### Assessment of high-quality studies based on Johnen et al.: Samples

Among the six reports classified with high quality according to the adapted scoring system by Johnen et al. [24] (see also Quality assessment: Canine scent detection work scores based on Johnen et al. and Table 2), axillary sweat [39], [42], arm crook sweat [45], [55], sweat from the head or face area or wrists [43], clothes [62], and saliva and urine [55] were used in the respective DTEs. None of the samples in the assessed DTEs of the six studies were inactivated. Sample acquisition time took from seconds (sweat from arm crooks, wrists, head or face area, and urine and saliva) to 2 minutes (axillary sweat) to 12 hours (clothes).

### Detection accuracy of dogs

**Fig. 5:**
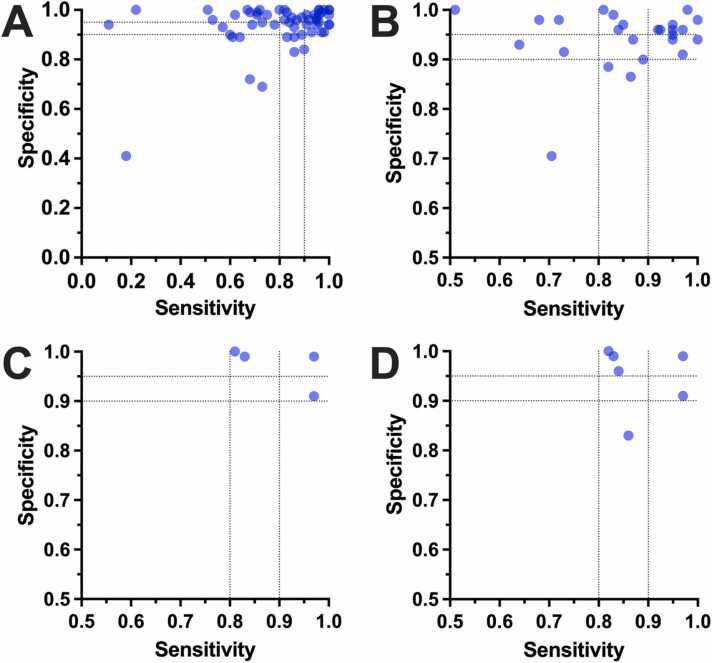
Scatter plots of assessed sensitivites (SENs) and specificities (SPEs) across reviewed studies and their diagnostic test evaluation trials (DTEs). (*A*) shows diagnostic performance of all assessed canine DTEs (n = 59; n = 56 DTEs are plotted due to missing exact SEN and/or SPE values in n = 3 DTEs; see also Supplementary Table 2). (*B*) summarizes diagnostic performance of DTEs per study (n = 27). If multiple DTEs were performed per study, the median for all DTEs per study was plotted. (*C*) shows the diagnostic performance of DTEs in the n = 4 studies with the least risk of bias according to the adapted QUADAS-2 assessment according to Whiting et al. [23]. Diagnostic performances of DTEs are plotted as median per study if multiple DTEs per study were selected for assessment. Please note that the studies may also be represented by only a subset of their DTEs (i.e., the selected DTEs; see also Quality assessment: QUADAS-2 for diagnostic accuracy studies, Table 1, and Supplementary Table 2). (*D*) shows the diagnostic performance of DTEs in the n = 6 studies with highest quality according to the adapted assessment system of canine scent detection performance of Johnen et al. [24]. Diagnostic performances of DTEs are plotted as median per study if multiple DTEs per study were selected for assessment. Please note that the studies may also be represented by only a subset of their DTEs (i.e., the selected DTEs; see also Quality assessment: Canine scent detection work scores based on Johnen et al., Table 2, and Supplementary Table 2). *Black horizontal lines* in (*A*–*D*) highlight 90% and 95% SPE, and *vertical black lines* highlight 80% and 90% SEN for better clarity, respectively. Please note furthermore that the axes in (*B*–*D*) start at SEN and SPE of 0.5, in contrast to (*A*).

Overall, 59 DTEs in 27 studies were conducted, with a median of two DTEs per study (range 1–8). Supplementary Table 1 provides calculation details and diagnostic metrics (SEN, SPE, and ACC) for each DTE. When the results of all conducted DTEs (Fig. 5*A*) were summarized per study as median, the range of SEN among studies (n = 27) was 51%–100%, with 78% of studies ≥80% SEN and 44% of studies ≥90% SEN. The range of SPE was 71%–100% with 88% of studies ≥90% SPE and 60% of studies ≥95% SPE (Fig. 5*B*). The range of ACC was 85%–100%, with 78% of studies ≥90% ACC and 48% of studies ≥95% ACC.

#### QUADAS-2 assessment of studies with least risk of bias: Detection performance

The four studies and their DTEs with the lowest risk of bias and applicability concerns according to QUADAS-2 [23] (Table 1) demonstrated 83% SEN, 99% SPE, and 99% ACC [39], 97% SEN, 91% SPE, and 93% ACC [42], 81% SEN, 100% SPE, and 100% ACC (medians of all DTEs of the study) [45], and 97% SEN, 99% SPE, and 98% ACC [43] (Fig. 5*C*). Latter study was represented by its second DTE (see also Table 1 and Supplementary Table 2).

#### Assessment of high-quality studies based on Johnen et al.: Detection performance

Taking the six reports [39], [42], [43], [45], [62], [55] and their DTEs into account, which showed high quality according to the adapted scoring system from Johnen et al. [24] (Table 2), the range of SEN across the six studies was 82%–97% with two studies showing ≥90% SEN. The range of SPE was 83%–100%, and five out of the six studies showed ≥90% SPE, whereas four out of six studies showed ≥95% SPE (Fig. 5*D*). The range of ACC was 84%–100%, with five out of six studies ≥90% ACC and three out of six studies ≥95% ACC. In the case of multiple DTEs per study, the diagnostic values of the respective study were represented by the selected DTE (or by the median performance of multiple DTEs per study if >1 DTE/study selected) according to the adapted system from Johnen et al. [24]. See also Quality assessment: Canine scent detection work scores based on Johnen et al., Table 2, and Supplementary Table 2 for more information.

## Discussion

The results of our systematic review show that *Canis lupus familiaris* is capable of detecting SARS-CoV-2 infections, by sniffing human biological material or individuals, with high SEN and SPE. When considering the selected DTEs of six studies with high quality according to the adapted scoring system of Johnen et al. [24], ranges of SEN and SPE reached 82%–97% and 83%–100%, respectively; in four studies, the SPE exceeded 95%. For the four studies and their DTEs with low risk of bias according to the QUADAS-2 tool [23], SENs ranged from 81% to 97% and SPEs from 91% to 100%. The results of the canine detection (index test) were compared to those obtained with RT-qPCR (reference standard), the gold standard method for detecting infection with SARS-CoV-2 (see also Supplementary Table 1 for diagnostic metrics and calculation procedures across all reviewed studies). It should be noted that, due to different processes in the collection of the results and partly missing information on the performance of individual dogs, a meta-analysis of the data was not performed, and the overall performance was presented as range.

A review by Dinnes et al. reported currently available point-of-care antigen tests providing SENs between 34.1% and 88.1% and an average SPE of 99.6% [66]. A meta-analysis by Khandker et al. revealed pooled SEN of 68.4% and SPE of 99.4% for rapid antigen tests in overall 17,171 suspected COVID-19 patients [67]. Another meta-analysis yielded a pooled antigen test SEN and SPE of 71.2% and 98.9%, respectively, by pooling 112,323 samples [68]. However, the minimal requirements for rapid antigen tests according to the Paul Ehrlich Institute (Langen, Germany) should exceed 80% of SEN and 97% of SPE [69]. The summarized results from this systematic review show that most SARS-CoV-2 canine scent detection is within or even above these requirements. In addition to the gold standard assumption of qRT-PCR, two of the reviewed studies performed Bayesian latent class analysis that also takes into account the imperfect status of a qRT-PCR test and allows for independent comparison between test systems [70]. Those studies showed that SEN and SPE of canine detection improved [39], [62], and the canine test is capable of outperforming the qRT-PCR with a SEN of 89% versus 73% while preserving high SPEs of 99% in both test systems [39].

Despite the good performance of dogs in discriminating SARS-CoV-2 samples from controls, the risks of bias and applicability concerns were remarkable among the studies (QUADAS-2 analysis; Table 1). This was particularly evident for the patient selection: only few noncase-controlled studies have been performed with testing individuals in SARS-CoV-2 screening centers [39], [42], homes for the elderly [41], health care and governmental sector, as well as public transport [40], [43], [44], and concerts [45]. Adequate patient selection for the validation of diagnostic accuracy studies is crucial. Observational studies may be biased since the adequate representation of the target population is questionable; this is due to the limited and quite targeted sample preparation and presentation, which can impact the interpretation of the respective diagnostic values [33]. Most studies have deliberately avoided high variability in the samples (see Tables 1 and 2) because they tested if dogs can, in principle, differentiate SARS-CoV-2 from healthy controls. Those can only be seen as pilot studies prior to testing dogs' performance in the field in public places.

In addition, other study design aspects to reduce bias were not considered in all studies (Table 2). Aspects such as blinding, randomization, novelty of samples, and sample repetitions in the DTEs are of great importance. A nonblinded or single-blinded design in canine scent detection studies (50% of reviewed studies) can impose a major risk for bias due to the sensible perception of dogs of unintentional and subtle cues from handlers or other present personnel (“Clever-Hans”-effect) [71]. Sample novelty and omission of sample repetitions during DTE are also important since generalization of the COVID-19-associated odor profile in the training procedure needs to be guaranteed, while recognition (or discrimination) of “personal” odor profiles needs to be avoided. In the case of infectious diseases, such as COVID-19, robust generalization is essential as the COVID-19 odor profile may vary depending on the course of the disease (asymptomatic, mild, and severe) and metabolic condition of the infected individual (age, sex, other diseases, and so on). Although there are initial chemoanalytical studies profiling COVID-19 VOCs [72], [73], [74], [75], [76], [77], [78], full characterization is still a long way off, if possible at all. For this reason, at an early stage of an endemic or pandemic, dogs should be trained with a varying range of samples with the same condition that, in the optimal case, are presented only once per dog. However, this also depends on the training method. In addition, in order to achieve robust discrimination between similar conditions (e.g., flu induced by influenza A virus versus COVID-19), other pathogens or diseases as negative controls should be involved in training procedures. In the case-controlled study by ten Hagen et al. dogs were able to discriminate between SARS-CoV-2 and 15 other pathogens inducing similar pathological conditions [57]. A slight decrease in detection performance compared to studies where samples from only healthy individuals were used as negative controls highlights the importance of implementing other pathogens or diseases into the training process. Furthermore, preliminary studies revealed differences in VOC profiles between influenza A and SARS-CoV-2 using laboratory-based methods [79], [80], which supports the olfactory discrimination ability of the dogs between similar pathological conditions.

Some studies did not exclude sample repetitions during DTEs, which was attributed to the sample shortage at the beginning of a pandemic [50] and methodological aspects (e.g., sample presentation method). Grandjean et al., using repetitive sample presentations in their DTE protocol, stated that dogs needed only 1 day sniffing between 4 and 10 positive samples during training in order to “learn” the odor [47]. Vesga et al. reported that only three samples (presented repeatedly) were enough to induce generalization [40]. The working group from Hannover, also using sample repetitions, documented each first reaction of the dogs to a new sample introduced into the test system; this ensured generalization before DTEs began [55], [57], [46]. Interestingly, Jendrny et al. could show a successful inter-sample type generalization, highlighting the evidence of a “universal” COVID-19 scent across human samples [55]. Essler et al. and Vlachová et al. used mixed samples in order to create “new” scents [50], [54], which makes estimation of impact on generalization difficult. Ultimately, how many different samples need to be presented prior to reaching generalization will depend on the target scent [30] and is currently unclear for SARS-CoV-2 detection. This lack of knowledge was reflected in a high variance of the total training duration across studies (a few days up to 4 months), which was mainly chosen arbitrarily and was partly based on a threshold of success in the training phase [43], [49]. To test if generalization for a target scent was successful, however, the presence of a “novelty cutoff” between training and DTE is considered very important since novel samples challenge the dogs, mimic real-life scenarios, and help to certainly exclude that dogs learned to detect personal odor profiles rather than common (disease-associated) odor profiles (Table 2). Research addressing sample presentation, training frequency, and duration for establishing optimal references can help to enhance standardization and avoid excessive generalization that may result in increased false positive alerts [30]. This highlights the need for a standardized “sensory balance” in order to be able to optimally deploy dogs as reliable medical detectors.

Further standardization challenges arise from the nature of the index test itself. Dogs are living beings with individual needs and different personality traits and experiences, which can be subject to ethical and animal welfare issues. In all reviewed studies, operant conditioning with positive reinforcement of correct searching behavior using reward (e.g., food and toy) was used [81], thus being ethically unobjectionable. However, aspects such as anatomy, physiology, physical and mental condition, age, sex, behavior, and extrinsic factors, such as experience and environment, can have an impact on olfaction and scent work performance [26], [82], [83]. In all studies, normocephalic dog breeds were involved, mainly Belgian Malinois, Labrador Retrievers, and German Shepherds, representing typical breeds involved in scent detection tasks (Fig. 2*C*). Brachycephalic breeds are supposed to have a lowered olfactory performance for anatomical and physiological reasons [36] and should be excluded for scent detection work [26]. Interestingly, performance in studies where dogs with varied experience levels were involved was similar among experienced and inexperienced (green) dogs [45], [51], [55], [57], [60], [63], [46], [49], [58]. There are indications that olfactory experience can be very beneficial for new olfactory tasks [27]. However, Chaber et al. revealed that experienced dogs (n = 7) had only slightly better accuracies in detection than green dogs (n = 8) [61]. This highlights the incorruptible speed with which dogs can be conditioned to a specific scent, masking long-term factors, such as previous olfactory experiences. Nevertheless, an additional training period for green dogs in order to understand and learn about COVID-19 searching contexts is crucial [49]. Only one study had deployed dogs with varying detection experience levels in such a large-scale screening scenario (music event) even though samples were provided in a line-up. Appropriate training preparation led to similar performance between experienced and inexperienced dogs, even if the latter only had experience with obedience training but no detection work [45]. However, in order to be able to ensure effective use of the canine olfactory potential, a high motivational drive and obedience of involved dogs is inevitable [26]. Frustration during scent detection work might be a problem, which should be avoided through the regular experience of success and reward, especially when the prevalence of the disease is low [45]. Therefore, it is also important to introduce blank trials during training, that is, without positive samples, in order to verify if dogs perform forced choices as trial-and-error due to high frustration levels. This procedure was reported by only a few studies [43], [44], [45], [62], [55], [57], [50], [61], [58]. Another consideration in comparison to antigen tests is that acquisition of learned content requires time, and extinction of this content occurs when no training is provided. Vesga et al. showed decreased sensitivities in dogs by 69% after a gap of training of 2.5 months and after introducing them to a new search context [40]. However, others could show that only a few days of retraining dogs to COVID-19 scent are sufficient to regain good performance [45], [55], [57], [64]. In summary, training and rapid retraining of dogs are safe, cost-effective, and time-saving options compared to other test developments. Although a canine testing speed of only seconds is decisive, issues of potential fatigue have to be considered. However, Guest et al. stated that two dogs can screen 300 people in 30 minutes [62], which exceeds the capacity of antigen tests by far.

Training paradigms across studies were only slightly different (Fig. 3; Supplementary Table 1). Mainly, the line-up approach was used. The group from Hannover used the DDTS device, which is mainly characterized by automated randomized and double-blinded sample presentations [45], [55], [57], [64], [46]. Although training periods were chosen arbitrarily across studies, it is to be assumed that automatic systems provide an optimized and quick workflow with fewer interventions by training personnel [84] and allow an uninfluenced interplay between dogs and machines leading to robust extinction of the “Clever-Hans”-effect [71]. Chaber et al., who used a double-blinded line-up setting, reported that dog handlers were not only blinded to the sample status but also to the run status [61]. That means, they did not know whether positive samples were present in a trial at all, which can fundamentally change the expectations of the handler. Automated systems during training and subsequent repurposing of the search contexts to line-up settings with double-blinded and randomized sample as well as run presentations (mimicking real-life scenarios) might represent a quite effective and time-saving method before deployment [45]. However, there is a lack of studies concerning canine training protocols for optimal searching purposes [85]. Training guidelines are currently under elaboration, and a detailed training protocol is provided as supplementary material by Chaber et al. [61].

Unlike the training methods, the used sample types and sample inactivation methods are variable among the studies (Fig. 4; Supplementary Table 1). Nevertheless, almost every study showed the high performance of involved dogs with high SENs and SPEs. This is not surprising since, in most of the studies, dogs were trained and tested with the same sample material (even if samples were novel in DTE). Interestingly, only a few studies have addressed the crucial question of whether transfer performance between the sample material and inactivation processes has an impact on the outcomes. Jendrny et al. showed that BPL inactivation does not influence performance when dogs were confronted with noninactivated samples afterward [55]. In addition, dogs were able to switch from saliva samples to urine and sweat samples with high SENs and SPEs, which shows that a broad “COVID-19 smell” is present in the infected individuals [55] and cell-culture supernatants [57]. In this context, subsequent studies from ten Hagen et al. and Twele et al. used different sample materials for training [45], [64]. Devillier et al. evaluated the performance switch from sweat samples (training) to worn surgical masks in the DTE [60]. Mancilla-Tapia et al. investigated sweat samples of different sources (axillary vs. corporal) [63]. Vesga et al. trained with saliva and tested dogs’ performance on respiratory samples in the first two DTEs [40]. Essler et al. investigated detergent and heat inactivation and transfer performance between saliva and urine with mixed results [50]. Those discrepancies might be due to BPL manipulating VOCs to a lesser extent (complete hydrolyzation) than heat exposure. Similarly, Demirbas et al. tested olfactory transfer between noninactivated masks and heat- and UV-inactivated masks, and noted some problems with the detection of heat-treated samples [65]. Noninactivated sweat samples were used in most studies due to practicability (quick and easy sampling) and safety concerns as sweat seems not to be a significant transmission pathway of SARS-CoV-2 [86], [87]. However, biosecurity aspects are of vital importance for protecting human and canine health. The susceptibility of dogs to SARS-CoV-2 is currently discussed controversially [88]. For this reason, research on the impact of inactivation methods and other safety measures (e.g., Training Aid Delivery Device containers) for safe sample presentation should be highlighted [55], [50]. Not every new emerging disease, in which dogs might be involved as detectors, is expected to have the same infection dynamics as SARS-CoV-2. Even SARS-CoV-2 shows different viral loads in different biological fluids over time [89], [90], which is a relevant safety concern. In addition, information about sample storage is versatile; for example, data on the duration of SARS-CoV-2 sample storage that allows the preservation of VOCs are not yet available. This highlights the need for optimization of the storage of biological samples for canine scent detection work, with the aim to establish robust training sample sets. Kantele et al. stated that they used storage time of sweat samples of 0–5 months for the validation trial (first DTE) with results of overall SEN of 92% and SPE of 91% [43]. One approach for enhancing standardization processes is to use animal models of SARS-CoV-2 [91] for sample generation, supernatants of cell cultures [57], or even viral proteins, which is currently under evaluation. However, aspects of adequate levels of generalization and discrimination are crucial here in the context of effective pandemic containment (e.g., detection of only certain virus variants by training with respective infected cell cultures or proteins). Therefore, a combination of differently trained dogs might be an acceptable approach for a repertoire of both broad and targeted canine screening methods.

The used assessment systems in this systematic review differ but were thought to be complementary to each other. QUADAS-2 [23] deals with the evaluation of diagnostic tests' accuracy and is a semiquantitative system created for systematic review purposes of laboratory diagnostic tests, whereas the system according to Johnen et al. [24] specifically emphasizes the quality of dogs’ olfactory work performance, highlighting possible confounding factors that could alter actual canine scent performance. In the latter case, the quality is measured via scores, and evaluating and contrasting the two dimensions, that is, a) diagnostic tests and b) olfactory work, can help to create a more holistic picture and to critically emphasize the potential of the dog as both a diagnostic tool and an olfactory detector, especially under “strict” or realistic conditions. Importantly, both systems were tailored and adapted to the review question concerning the potential of dogs for screening a disease in a human population (see Assessment of quality of diagnostic accuracy studies–QUADAS-2 and Quality scoring of canine scent detection work based on Johnen et al.). Especially, the system of Johnen et al. [24] was slightly modified to match the review question in terms of medical scent detection, and scores were adjusted accordingly. The arbitrary weighting and balancing of the scores are based not only on the study of Johnen et al. [24] but also on tailoring to the review question, conscientious research work, empirical values, and expert opinions. Nevertheless, a certain bias in the evaluation using arbitrary scores can never be excluded. Some studies highlight that scoring systems might not be adequate for the appropriate assessment of study quality [25], [92]. However, it is highlighted that both evaluation systems were used independently, and it was, furthermore, noticeable that all studies with the least risk of bias according to the QUADAS-2 system also appear among the high-quality studies according to the scoring system of Johnen et al. [24], which suggests a certain level of consensus. A further limitation is that one of the quality assessing authors (SM) is also co-author in various of the assessed studies and was the only author who led and performed the study searching and identification step. Search terms were not defined by a trained librarian and were only determined by the co-authors. Unpublished articles that entered the study selection and quality assessment process were mainly provided by the detection dog community via personal communication in order to provide a comprehensive and up-to-date analysis. Other authors did check the list of identified studies but did not perform a detailed search as SM, so studies could have been missed. Study identification and quality assessment were performed very conscientiously and according to objective standards based on the tools used in consultation with the statistician co-authors (see also Material and methods); however, there is a risk of bias when only one person evaluates the evidence. Nevertheless, the quality aspects were endorsed by all authors who are experts in the field and in statistics.

The high diagnostic values and, at the same time, the incomparable velocity of the canine detection of only seconds clearly show that dogs could be valuable for detecting infectious diseases as a supportive measure, both for the current COVID-19 pandemic and potentially for future epidemics and pandemics. Although only 4–6 studies out of 27 studies showed a low risk of bias and high study quality, respectively, it highlights that the canine olfactory system fulfills many criteria of an ideal screening test, including quick performance, high accuracy, safety, practicability, noninvasiveness, and cost-effectiveness, while it can also be used in the field [33]. However, different aspects, such as canine breeds, training paradigms, training duration, sample type, or sample preprocessing (e.g., inactivation), do need to be considered in terms of study planning and timing, logistics, safety measurements, and so on for possible and efficient deployment of dogs. Thus, when considering canine medical scent detection as a screening test, those mentioned aspects should be standardized, and dog’s and handler’s performance need to be certified and recertified on a regular basis using a similar procedure as with canine detection of explosives [28].

## Conclusions

Prior to the flurry of new research into canine medical detection of SARS-CoV-2, multiple former studies showed that dogs are able to detect human diseases, infectious and even noninfectious in principle. As with any test system, standardization and certification procedures are necessary to ensure that promising results in a laboratory setting can be achieved in real-life testing scenarios. Legal frameworks and guidelines exist for the detection of explosives, and it would need the regulators to develop similar guidelines for medical detection dogs.

There is relatively solid evidence for dogs to detect samples from SARS-CoV-2-infected individuals in a laboratory setting, but there are only less of a handful studies that have shown the successful use of dogs in the field. There are many aspects that distinguish the canine nose test from the conventionally available, industrially produced tests, including the fact that dogs are living beings with their own needs and wills, different personalities and capabilities, as well as physical limitations. This must be respected, which inevitably leads to ethical and animal welfare considerations and challenges for standardization. Canine breeds and personalities, training methods, acquisition, preparation, and presentation of samples, environmental influences, safety considerations, and so on must be subject to ethical and animal welfare-related scrutiny and necessary standardization processes.

However, as in explosives detection work, dogs do have their merit for screening for infectious diseases, especially in remote places. Dogs may be of particular importance for countries and regions where a solid testing and vaccination infrastructure cannot be established for various reasons. Public spaces and crowds of people sensitive to pandemic dynamics (schools, airports, events, and so on) can greatly benefit from the speed of canine detection. Although dogs are not, and should not be, the sole means of pandemic control, they can provide an additional and relatively cost-effective testing strategy.

## Author contributions

HAV, FT, CC, FOM, and SM discussed and planned the form of the systematic review and of the analyses to be conducted. SM wrote the manuscript and conducted the main data extraction and analyses. MC and SM conducted the QUADAS-2 assessment. CS and LK gave statistical support and helped with the planning of the result presentation. A-LC, LD, DG, FOM, SR, and all other mentioned authors have contributed significantly to the completion and development of the review with relevant expertise. All authors have read and approved the final manuscript.

## Declaration of Competing Interest

The authors declare that they have no known competing financial interests or personal relationships that could have appeared to influence the work reported in this paper.

## Footnotes

Appendix A Supplementary data associated with this article can be found in the online version at doi:10.1016/j.annepidem.2023.05.002.
