# Supplementary material for: Canine olfactory detection of SARS-CoV-2-infected humans—a systematic review
Source: Ann Epidemiol. 2023 Sep;85:68–85. doi: 10.1016/j.annepidem.2023.05.002 (PMC10195768; doi:10.1016/j.annepidem.2023.05.002)
Supplement: Supplementary file 1 — Supplementary material [file mmc1.docx]

| **general** | | | **dogs** | | | **samples** | | | | | **training** | | | | | | **diagnostic test evaluation (DTE)** | | | | | | | **results (diagnostic metrics for DTEs)** | | | | |
| --- | --- | --- | --- | --- | --- | --- | --- | --- | --- | --- | --- | --- | --- | --- | --- | --- | --- | --- | --- | --- | --- | --- | --- | --- | --- | --- | --- | --- |
| **report* [ref]** | **country** | **study design** | **N** | **skull anatomy** | **previous experience in scent work?** | **multiple sample sources/ variation for [+] and [-] samples included (for DTE)?** | **Different stages (symptomatic/ asymptomatic) of COVID-19 samples included**  **(for DTE)?** | **Other diseases, symptoms, or pathogens in [-] samples included (for DTE)?** | **Sample acquisition time for DTE** | **Storage of samples for DTE** | **used scenario** | **duration** | **[+] & [-]**  **sample type (inactivation)** | **approximate sum of positive [+] and negative [-] sample presentations (all dogs)** | | **repeated presentations in at least one dog?** | **used scenario** | **[+] & [-]**  **sample type (inactivation type)** | **approximate sum of positive [+] and negative [-] sample presentations (all dogs)** | | **single blind (b), double blind (db), and/or randomized (r) sample presentations?** | **repeated sample presentations in at least one dog?** | **mocks and “empty” samples included in diagnostic metrics?** | **proportion of positive sample presentations** | **sensitivity (SEN)** | **specificity (SPE)** | **accuracy (ACC)** | **further information about assessment of diagnostic metrics** |
|  |  |  |  |  |  |  |  |  |  |  |  |  |  | **[+]** | **[-]** |  |  | **Mock or “empty” samples included?** | **[+]** | **[-]** | **novelty of samples in DTE guaranteed?** |  |  |  | **range (R) & interquartile range (IQR)** | **range (R) & interquartile range (IQR)** | **range (R) & interquartile range (IQR)** |  |
|  |  |  |  |  |  |  |  |  |  |  |  |  |  | **definition of sample status (reference)** | |  |  |  | **definition of sample status (reference)** | |  |  |  |  |  |  |  |  |

**Supplementary Table 1** Detailed information and performance overview of the evaluated studies

| Jendrny et al., 2020 [46] | Germany | case | 8 | normo-cephalic | varied | yes | no | *unclear* | seconds | min. 7 d before dog contact | DDTS | 2 w (habit.);  1 w (impr.) | [+] & [-]:  saliva & tracheobronchial secretions (BPL) | 1484 | 8904 | yes | DDTS | [+] & [-]:  saliva & tracheobronchial secretions (BPL) | 190 | 822 | db & r | yes, included in diagnostic metrics | - | 0.19 | 0.85 | 0.97 | 0.95 | median of all dogs per DTE; additional metrics calculated from published results |
| --- | --- | --- | --- | --- | --- | --- | --- | --- | --- | --- | --- | --- | --- | --- | --- | --- | --- | --- | --- | --- | --- | --- | --- | --- | --- | --- | --- | --- |
|  |  |  |  |  |  |  |  |  |  |  |  |  |  | by PCR of participant | |  |  | no | by PCR of participant | | yes |  |  |  | R: 0.7–0.95  IQR: 0.73–0.91 | R: 0.92–0.99  IQR: 0.95–0.985 | R: 0.87–0.97  IQR: 0.92–0.968 |  |

| Grandjean et al., 2020 [47] | France | case | 14 (train.)  6 (DTE) | normo-cephalic | yes | yes | no | no | 20 min | gauze, polymer tube; cooled; min. 24 h before dog contact | line-up | 1–3 w (overall) | [+] & [-]:  axillary sweat (none) | *unclear* | *unclear* | line-up | [+] & [-]:  axillary sweat (none) | approx. 334 | approx. 780 | b & r | yes, included in diagnostic metrics | no | approx. 0.3 | 0.95 | 0.96 | 0.96 | median of all dogs per DTE; additional metrics calculated from published supplementary material (w/o mocks) |
| --- | --- | --- | --- | --- | --- | --- | --- | --- | --- | --- | --- | --- | --- | --- | --- | --- | --- | --- | --- | --- | --- | --- | --- | --- | --- | --- | --- |
|  |  |  |  |  |  |  |  |  |  |  |  |  |  | by PCR of participant |  |  | yes | by PCR of participant | | yes |  |  |  | R: 0.83–1.0  IQR: 0.85–1.0 | R: 0.93–1.0  IQR: 0.94–1.0 | R: 0.9–1.0  IQR: 0.9–1.0 |  |

| Eskandari et al., 2021 [48] |  | | | | | | | | | | | | | | | | | | | | | | | | | | |
| --- | --- | --- | --- | --- | --- | --- | --- | --- | --- | --- | --- | --- | --- | --- | --- | --- | --- | --- | --- | --- | --- | --- | --- | --- | --- | --- | --- |
| – DTE 1 | Iran | case | 3 | normo-cephalic | *unclear* | yes | yes | *unclear* | seconds | *unclear* | scent wheel | 7 w (overall) | [+] & [-]:  nasopharyngeal secretions (none) | *unclear* | *unclear* | *unclear* | [+] & [-]:  nasopharyngeal secretions (none) | 26 | 54 | b  r: *unclear* | no | - | 0.33 | 0.6 | 0.9 | 0.8 | median of all dogs per DTE; additional metrics calculated from published results |
|  |  |  |  |  |  |  |  |  |  |  |  |  |  | by PCR of participant |  |  | no | by PCR of participant | | *unclear* |  |  |  | R: 0.6–0.83  IQR: 0.6–0.83 | R: 0.85–0.93  IQR: 0.85–0.93 | R: 0.77–0.9  IQR: 0.77–0.9 |  |
| – DTE 2 | Iran | case | 3 | normo-cephalic | *unclear* | yes | yes | *unclear* | 24 h | *unclear* | scent wheel | 7 w (overall) | [+] & [-]:  clothes & masks (none) | *unclear* *(*approx. 1300 clothes & masks overall) | *unclear* | *unclear* | [+] & [-]:  clothes & masks (none) | 50 | 70 | b  r: *unclear* | no | - | 0.42 | 0.86 | 0.93 | 0.9 | overall (one general contingency table per DTE); additional metrics calculated from published results |
|  |  |  |  |  |  |  |  |  |  |  |  |  |  | by PCR of participant |  |  | no | by PCR of participant | | *unclear* |  |  |  | - | - | - |  |

| Grandjean et al., 2021 [49] | UAE | case | 21 | normo-cephalic | varied | yes | no | yes | ≤ 1–20 min | polymer tube; cooled; min. 24 h before dog contact | line-up | ≥ 2 w (overall) | [+] & [-]:  axillary sweat (none) | 1786 (overall) | *unclear* | line-up | [+] & [-]:  axillary sweat (none) | 769 | min. 879 | b & r | yes, but for [-] samples only, therefore irrelevant since only SEN available. | no | *unclear* | 0.93 | - | - | median of all dogs per DTE |
| --- | --- | --- | --- | --- | --- | --- | --- | --- | --- | --- | --- | --- | --- | --- | --- | --- | --- | --- | --- | --- | --- | --- | --- | --- | --- | --- | --- |
|  |  |  |  |  |  |  |  |  |  |  |  |  |  | by PCR of participant |  |  | yes | by PCR of participant | | yes |  |  |  | R: 0.71–1.0  IQR: 0.87–0.99 |  |  |  |

| Essler et al., 2021 [50] |  | | | | | | | | | | | | | | | | | | | | | | | | | | | |
| --- | --- | --- | --- | --- | --- | --- | --- | --- | --- | --- | --- | --- | --- | --- | --- | --- | --- | --- | --- | --- | --- | --- | --- | --- | --- | --- | --- | --- |
| – DTE 1 | USA | case | 9 | normo-cephalic | *unclear* | yes | *unclear* | *unclear* | minutes | cotton ball, sample cups; frozen; samples mixed | single stand/ scent wheel | > 3.5 w (overall) | [+] & [-]:  urine (detergent) | *unclear* | | yes | scent wheel | [+] & [-]:  urine (detergent) | min. 126 | min. 504 | *unclear* | yes & samples mixed (might potentially impact also first pass metrics) | no | 0.2 (first pass) | 0.71 | 0.99 | 0.94 | overall (one general contingency table per DTE) |
|  |  |  |  |  |  |  |  |  |  |  |  |  |  | by PCR of participant | |  |  | *unclear* | by PCR or antigen test of participant | | *unclear* |  |  |  | - | - | - |  |
| – DTE 2 | USA | case | 8 | normo-cephalic | *unclear* | yes | *unclear* | *unclear* | minutes | cotton ball, sample cups; frozen; samples mixed | single stand/ scent wheel | > 3.5 w (overall) | [+] & [-]:  urine (detergent) | *unclear* | | yes | scent wheel | [+]: urine (heat)  [-]: urine (detergent) | min. 8 | min. 32 | *unclear* | yes & samples mixed (might potentially impact also first pass metrics) | no | 0.2 (first pass) | 0.75 | 0.98 | 1.0 |  |
|  |  |  |  |  |  |  |  |  |  |  |  |  |  | by PCR of participant | |  |  | *unclear* | by PCR or antigen test of participant | | no |  |  |  | - | - | - |  |
| – DTE 3 | USA | case | 8 | normo-cephalic | *unclear* | yes | *unclear* | *unclear* | minutes | cotton ball, sample cups; frozen; samples mixed | single stand/ scent wheel | > 3.5 w (overall) | [+] & [-]:  urine (detergent) | *unclear* | | yes | scent wheel | [+] & [-]:  urine (heat) | min. 8 | min. 32 | *unclear* | yes & samples mixed (might potentially impact also first pass metrics) | no | 0.2 (first pass) | 0.62 | 0.98 | 0.62 |  |
|  |  |  |  |  |  |  |  |  |  |  |  |  |  | by PCR of participant | |  |  | *unclear* | by PCR or antigen test of participant | | yes |  |  |  | - | - | - |  |
| – DTE 4 | USA | case | 9 | normo-cephalic | *unclear* | yes | *unclear* | *unclear* | minutes | cotton ball, sample cups; frozen; samples mixed | single stand/ scent wheel | *unclear* | [+] & [-]:  urine (heat) | *unclear* | | yes | scent wheel | [+] & [-]:  urine (heat) | min. 126 | min. 486 | *unclear* | yes & samples mixed (might potentially impact also first pass metrics) | no | 0.21 (first pass) | 0.71 | 0.98 | 0.94 |  |
|  |  |  |  |  |  |  |  |  |  |  |  |  |  | by PCR of participant | |  |  | *unclear* | by PCR or antigen test of participant | | *unclear* |  |  |  | - | - | - |  |
| – DTE 5 | USA | case | 9 | normo-cephalic | *unclear* | yes | *unclear* | *unclear* | minutes | cotton ball, sample cups; frozen; samples mixed | single stand/ scent wheel | *unclear* | [+] & [-]:  urine (heat) | *unclear* | | yes | scent wheel | [+] & [-]:  urine (heat) | min. 45 | min. 180 | b  r: *unclear* | yes & samples mixed (might potentially impact also first pass metrics) | no | 0.2 (first pass) | 0.68 | 0.99 | 0.96 |  |
|  |  |  |  |  |  |  |  |  |  |  |  |  |  | by PCR of participant | |  |  | *unclear* | by PCR or antigen test of participant | | no |  |  |  | - | - | - |  |
| **general** | | | **dogs** | | | **samples** | | | | | **training** | | | | | | **diagnostic test evaluation (DTE)** | | | | | | | **results (diagnostic metrics for DTEs)** | | | | |
| **report* [ref]** | **country** | **study design** | **N** | **skull anatomy** | **previous experience in scent work?** | **multiple sample sources/ variation for [+] and [-] samples included (for DTE)?** | **Different stages (symptomatic/ asymptomatic) of COVID-19 samples included**  **(for DTE)?** | **Other diseases, symptoms, or pathogens in [-] samples included (for DTE)?** | **Sample acquisition time for DTE** | **Storage of samples for DTE** | **used scenario** | **duration** | **[+] & [-]**  **sample type (inactivation)** | **approximate sum of positive [+] and negative [-] sample presentations (all dogs)** | | **repeated presentations in at least one dog?** | **used scenario** | **[+] & [-]**  **sample type (inactivation type)** | **approximate sum of positive [+] and negative [-] sample presentations (all dogs)** | | **single blind (b), double blind (db), and/or randomized (r) sample presentations?** | **repeated sample presentations in at least one dog?** | **mocks and “empty” samples included in diagnostic metrics?** | **proportion of positive sample presentations** | **sensitivity (SEN)** | **specificity (SPE)** | **accuracy (ACC)** | **further information about assessment of diagnostic metrics** |
|  |  |  |  |  |  |  |  |  |  |  |  |  |  | **[+]** | **[-]** |  |  | **Mock or “empty” samples included?** | **[+]** | **[-]** | **novelty of samples in DTE guaranteed?** |  |  |  | **range (R) & interquartile range (IQR)** | **range (R) & interquartile range (IQR)** | **range (R) & interquartile range (IQR)** |  |
|  |  |  |  |  |  |  |  |  |  |  |  |  |  | **definition of sample status (reference)** | |  |  |  | **definition of sample status (reference)** | |  |  |  |  |  |  |  |  |

**Supplementary Table 1** Detailed information and performance overview of the evaluated studies *(continued)*

| Essler et al., 2021 [50] *(continued)* |  | | | | | | | | | | | | | | | | | | | | | | | | | | | |
| --- | --- | --- | --- | --- | --- | --- | --- | --- | --- | --- | --- | --- | --- | --- | --- | --- | --- | --- | --- | --- | --- | --- | --- | --- | --- | --- | --- | --- |
| – DTE 6 | USA | case | 8 | normo-cephalic | *unclear* | yes | *unclear* | *unclear* | minutes | cotton ball, sample cups; frozen; samples mixed | single stand/ scent wheel | *unclear* | [+] & [-]:  urine (heat) | *unclear* | yes | scent wheel | [+] & [-]:  urine (heat) | min. 16 | min. 96 | | b  r: *unclear* | yes & samples mixed (might potentially impact also first pass metrics) | no | 0.14 (first pass) | 0.18 | 0.41 | 0.11 | overall (one general contingency table per DTE) |
|  |  |  |  |  |  |  |  |  |  |  |  |  |  | by PCR of participant |  |  | *unclear* | by PCR or antigen test of participant | | | yes |  |  |  | - | - | - |  |
| – DTE 7 | USA | case | 9 | normo-cephalic | *unclear* | yes | *unclear* | *unclear* | seconds | cotton ball, sample cups; frozen; samples mixed | single stand/ scent wheel | *unclear* | [+] & [-]:  urine (heat) | *unclear* | yes | scent wheel | [+] & [-]:  saliva (heat) | min. 9 | min. 36 | | *unclear* | yes & samples mixed (might potentially impact also first pass metrics) | no | 0.2 (first pass) | 0.22 | 1.0 | 0.67 |  |
|  |  |  |  |  |  |  |  |  |  |  |  |  |  | by PCR of participant |  |  | *unclear* | by PCR or antigen test of participant | | | no |  |  |  | - | - | - |  |
| – DTE 8 | USA | case | 9 | normo-cephalic | *unclear* | yes | *unclear* | *unclear* | seconds | cotton ball, sample cups; frozen; samples mixed | single stand/ scent wheel | *unclear* | [+] & [-]:  urine (heat) | *unclear* | yes | scent wheel | [+] & [-]:  saliva (heat) | min. 9 | | min. 36 | *unclear* | yes & samples mixed (might potentially impact also first pass metrics) | no | 0.2 (first pass) | 0.11 | 0.94 | 1.0 |  |
|  |  |  |  |  |  |  |  |  |  |  |  |  |  | by PCR of participant |  |  | *unclear* | by PCR or antigen test of participant | | | yes |  |  |  | - | - | - |  |

| Hag-Ali et al., 2021 [39] | UAE | cross | 4 | normo-cephalic | yes | yes | yes | yes | *unclear* | gauze; cooled (train.) &  room temperature (DTE); max. 2 h before dog contact | line-up | 1 d (habit.); 5.5 w (impr.) | [+] & [-]:  axillary sweat (none) | *unclear,* origin. 234 samples | *unclear,* origin. 144 samples | yes | line-up | [+] & [-]:  axillary sweat (none) | multiple dogs provided a unique status for each sample (origin. n=18) | multiple dogs provided a unique status for each sample (origin. n=3272) | db & r | no | - | 0.005  (based on sample count, not on sample status definition by dogs) | 0.83 | 0.99 | 0.99 | overall (one general contingency table per DTE); detections of multiple dogs of the same sample defined sample status and metrics; additional metrics calculated from published results |
| --- | --- | --- | --- | --- | --- | --- | --- | --- | --- | --- | --- | --- | --- | --- | --- | --- | --- | --- | --- | --- | --- | --- | --- | --- | --- | --- | --- | --- |
|  |  |  |  |  |  |  |  |  |  |  |  |  |  | by PCR of participant | |  |  | no | by PCR of participant | | yes |  |  |  | - | - | - |  |

| Mendel et al., 2021 [51] |  | | | | | | | | | | | | | | | | | | | | | | | | | | |
| --- | --- | --- | --- | --- | --- | --- | --- | --- | --- | --- | --- | --- | --- | --- | --- | --- | --- | --- | --- | --- | --- | --- | --- | --- | --- | --- | --- |
| – DTE 1 | USA | case | 4 | normo-cephalic | varied | no | *unclear* | yes | 30–40 min | min. 24–72 h before dog contact | scent wheel | > 1 month (overall) | [+]: worn masks (UV)  [-]: worn masks (*unclear)* | *unclear* | *unclear* | scent wheel | [+]: worn masks (UV)  [-]: worn masks (*unclear*) | approx. 140 | approx. 651 | b: *unclear*  r | yes, included in diagnostic metrics | - | approx. 0.18 | 0.61 | 0.89 | 0.83 | median of all dogs per DTE; additional metrics calculated from published results |
|  |  |  |  |  |  |  |  |  |  |  |  |  |  | by PCR of participant |  |  | no | by PCR of participant | | *unclear* |  |  |  | R: 0.56–0.86  IQR: 0.563–0.81 | R: 0.88–0.97  IQR: 0.883–0.95 | R: 0.8–0.96  IQR: 0.803–0.93 |  |
| – DTE 2 | USA | case | 4 | normo-cephalic | varied | no | *unclear* | *controls were unused masks* | 30–40 min | min. 24–72 h before dog contact | *training for DTE 2 = training for DTE 1 and DTE 1 itself* | | | | | scent wheel | [+]: worn masks (UV)  [-]: unused masks (*unclear*) | approx. 117 | approx. 557  (unused masks) | b: *unclear*  r | yes, included in diagnostic metrics | yes | approx. 0.17 | 0.91 | 0.98 | 0.97 |  |
|  |  |  |  |  |  |  |  |  |  |  |  |  |  |  |  |  | yes | by PCR of participant | - | no |  |  |  | R: 0.84–0.95  IQR: 0.86–0.94 | R: 0.95–0.99  IQR: 0.955–0.988 | R: 0.94–0.97  IQR: 0.95–0.97 |  |
| – DTE 3 | USA | case | 4 | normo-cephalic | varied | no | *unclear* | *unclear* | 30–40 min | min. 24–72 h before dog contact | *Training for DTE 3 = training for DTEs 1 & 2 (and DTEs 1 & 2 themselves)* | | | | | scent wheel | [+]: worn masks (UV); [-]: masks from ‘healthy persons’ & unused (*unclear*) | approx. 227 | approx. 5367  *(unclear if empty containers and unused masks included)* | b & r (*unclear if db since no info about status of other present persons*) | yes, included in diagnostic metrics | *unclear* | approx. 0.04 | 0.72 | 1.0 | 0.97 |  |
|  |  |  |  |  |  |  |  |  |  |  |  |  |  |  |  |  | yes | by PCR of participant | *unclear how ’healthy persons’ is defined* | no |  |  |  | R: 0.67–0.73  IQR: 0.68–0.73 | R: 0.99–1.0  IQR: 0.99–1.0 | R: 0.96–0.99  IQR: 0.96–0.988 |  |

| Angeletti et al., 2021 [52] | Italy | case | 3 | normo-cephalic | yes | no,  [+] & [-] samples both from one and the same facility each | no | yes | 5 min | gauze; cooled; max. 24 h before dog contact | single stand/ line-up | 4 w (overall) | [+] & [-]:  axillary sweat (none) | *unclear* | yes | line-up | [+] & [-]:  axillary sweat (none) | *unclear* | b: *unclear*  r | *unclear* | no | *unclear* | 0.87  (correct trials) | - | - | median of all dogs per DTE; SEN is considered percentage of correct trials in this case |
| --- | --- | --- | --- | --- | --- | --- | --- | --- | --- | --- | --- | --- | --- | --- | --- | --- | --- | --- | --- | --- | --- | --- | --- | --- | --- | --- |
|  |  |  |  |  |  |  |  |  |  |  |  |  |  | *unclear* |  |  | yes | *unclear* | no |  |  |  | R: 0.85–0.88  IQR: 0.85–0.88 | - | - |  |

| **general** | | | **dogs** | | | **samples** | | | | | **training** | | | | | | **diagnostic test evaluation (DTE)** | | | | | | | **results (diagnostic metrics for DTEs)** | | | | |
| --- | --- | --- | --- | --- | --- | --- | --- | --- | --- | --- | --- | --- | --- | --- | --- | --- | --- | --- | --- | --- | --- | --- | --- | --- | --- | --- | --- | --- |
| **report* [ref]** | **country** | **study design** | **N** | **skull anatomy** | **previous experience in scent work?** | **multiple sample sources/ variation for [+] and [-] samples included (for DTE)?** | **Different stages (symptomatic/ asymptomatic) of COVID-19 samples included**  **(for DTE)?** | **Other diseases, symptoms, or pathogens in [-] samples included (for DTE)?** | **Sample acquisition time for DTE** | **Storage of samples for DTE** | **used scenario** | **duration** | **[+] & [-]**  **sample type (inactivation)** | **approximate sum of positive [+] and negative [-] sample presentations (all dogs)** | | **repeated presentations in at least one dog?** | **used scenario** | **[+] & [-]**  **sample type (inactivation type)** | **approximate sum of positive [+] and negative [-] sample presentations (all dogs)** | | **single blind (b), double blind (db), and/or randomized (r) sample presentations?** | **repeated sample presentations in at least one dog?** | **mocks and “empty” samples included in diagnostic metrics?** | **proportion of positive sample presentations** | **sensitivity (SEN)** | **specificity (SPE)** | **accuracy (ACC)** | **further information about assessment of diagnostic metrics** |
|  |  |  |  |  |  |  |  |  |  |  |  |  |  | **[+]** | **[-]** |  |  | **Mock or “empty” samples included?** | **[+]** | **[-]** | **novelty of samples in DTE guaranteed?** |  |  |  | **range (R) & interquartile range (IQR)** | **range (R) & interquartile range (IQR)** | **range (R) & interquartile range (IQR)** |  |
|  |  |  |  |  |  |  |  |  |  |  |  |  |  | **definition of sample status (reference)** | |  |  |  | **definition of sample status (reference)** | |  |  |  |  |  |  |  |  |

**Supplementary Table 1** Detailed information and performance overview of the evaluated studies *(continued)*

| Sarkis et al., 2022 [53] | Lebanon | case | 2 | normo-cephalic | no | yes | yes | no | *unclear* | gauze; cooled | line-up | 1 m (overall) | [+] & [-]:  axillary sweat (none) | *unclear* | *unclear* | line-up | [+] & [-]:  axillary sweat (none) | 512 | approx. 406 | b & r | no | no | approx. 0.56 | 1.0 | 0.94 | 0.975 | median of all dogs per DTE; additional metrics calculated from published results |
| --- | --- | --- | --- | --- | --- | --- | --- | --- | --- | --- | --- | --- | --- | --- | --- | --- | --- | --- | --- | --- | --- | --- | --- | --- | --- | --- | --- |
|  |  |  |  |  |  |  |  |  |  |  |  |  |  | by PCR of participant |  |  | *unclear* | by PCR of participant | | yes |  |  |  | - | R: 0.92–0.88  IQR: 0.85–0.88 | R: 0.97–0.98  IQR: 0.97–0.98 |  |

| Vlachová et al., 2021 (*preprint*) [54] | Czech Republic | case | 4 (train.)  2 (DTE) | normo-cephalic | yes | no | *unclear* | *unclear* | sweat (torso):  20 min  breath: 3 min | room temperature; min. 24 h before dog contact; samples mixed | line-up | 14 d (impr.) | [+] & [-]:  sweat torso/ breath (none) | *unclear* | yes | line-up | [+] & [-]:  sweat torso/ breath (none) | 100 | 100 | b & r | *unclear* (samples mixed, might potentially impact also first pass metrics) | no | 0.5 | 0.95 | 0.94 | 0.945 | median of all dogs per DTE; additional metrics calculated from published results |
| --- | --- | --- | --- | --- | --- | --- | --- | --- | --- | --- | --- | --- | --- | --- | --- | --- | --- | --- | --- | --- | --- | --- | --- | --- | --- | --- | --- |
|  |  |  |  |  |  |  |  |  |  |  |  |  |  | by PCR of participant |  |  | yes | by PCR of participant | | *unclear for [-]* |  |  |  | R: 0.94–0.96  IQR: 0.94–0.96 | R: 0.92–0.96  IQR: 0.92–0.96 | R: 0.94–0.95  IQR: 0.94–0.95 |  |

| Jendrny et al., 2021 [55] |  | | | | | | | | | | | | | | | | | | | | | | | | | | |
| --- | --- | --- | --- | --- | --- | --- | --- | --- | --- | --- | --- | --- | --- | --- | --- | --- | --- | --- | --- | --- | --- | --- | --- | --- | --- | --- | --- |
| – DTE 1 | Germany | case | 10 | normo-cephalic | varied, some COVID-19 experience | yes | yes | yes | seconds | sample tube; frozen | DDTS | 6 d (habit.);  8 d (impr.) | [+] & [-]:  saliva (BPL) | *unclear* | yes | DDTS | [+] & [-]:  saliva (none) | 193 | 874 | db & r | yes, included in diagnostic metrics | - | 0.18 | 0.84 | 0.95 | 0.93 | median of all dogs per DTE |
|  |  |  |  |  |  |  |  |  |  |  |  |  |  | by PCR of participant |  |  | no | by PCR of participant | | yes |  |  |  | R: 0.61–1.0  IQR: 0.71–0.93 | R: 0.82–1.0  IQR: 0.94–0.96 | R: 0.81–0.97  IQR: 0.9–0.95 |  |
| – DTE 2 | Germany | case | 10 | normo-cephalic | varied, some COVID-19 experience | yes | yes | yes | seconds–minutes | sample tube, cotton pad; frozen | DDTS | 6 d (habit.);  8 d (impr.) | [+] & [-]:  saliva (BPL) | *unclear* | yes | DDTS | [+] & [-]:  saliva, arm crook sweat, urine (none) | 282 | 1388 | db & r | yes, included in diagnostic metrics | - | 0.17 | 0.53 | 0.96 | 0.9 |  |
|  |  |  |  |  |  |  |  |  |  |  |  |  |  | by PCR of participant |  |  | no | by PCR of participant | | yes |  |  |  | R: 0.61–1.0  IQR: 0.71–0.93 | R: 0.88–0.99  IQR: 0.93–0.99 | R: 0.84–0.97  IQR: 0.87–0.91 |  |
| – DTE 3 | Germany | case | 9 | normo-cephalic | varied, some COVID-19 experience | yes | yes | yes | seconds | cotton pad; frozen | DDTS | 6 d (habit.);  8 d (impr.) | [+] & [-]:  saliva (BPL) | *unclear* | yes | DDTS | [+] & [-]:  arm crook sweat (none) | 113 | 418 | db & r | yes, included in diagnostic metrics | - | 0.21 | 0.91 | 0.94 | 0.93 |  |
|  |  |  |  |  |  |  |  |  |  |  |  |  |  | by PCR of participant |  |  | no | by PCR of participant | | yes |  |  |  | R: 0.56–1.0  IQR: 0.71–0.96 | R: 0.88–1.0  IQR: 0.91–0.98 | R: 0.85–0.96  IQR: 0.88–0.95 |  |
| – DTE 4 | Germany | case | 10 | normo-cephalic | varied, some COVID-19 experience | yes | yes | yes | seconds–minutes | sample tube; frozen | DDTS | 6 d (habit.);  8 d (impr.) | [+] & [-]:  saliva (BPL) | *unclear* | yes | DDTS | [+] & [-]:  urine (none) | 117 | 477 | db & r | yes, included in diagnostic metrics | - | 0.2 | 0.96 | 0.98 | 0.95 |  |
|  |  |  |  |  |  |  |  |  |  |  |  |  |  | by PCR of participant |  |  | no | by PCR of participant | | yes |  |  |  | R: 0.5–1.0  IQR: 0.75–1.0 | R: 0.93–1.0  IQR: 0.95–1.0 | R: 0.88–1.0  IQR: 0.94–0.99 |  |
| – DTE 5 | Germany | case | 10 | normo-cephalic | varied, some COVID-19 experience | yes | yes | yes | seconds | sample tube; frozen | DDTS | 6 d (habit.);  8 d (impr.) | [+] & [-]:  saliva (BPL) | *unclear* | yes | DDTS | [+] & [-]:  saliva (none) | 249 | 1197 | db & r | yes, included in diagnostic metrics | - | 0.17 | 0.82 | 0.96 | 0.94 |  |
|  |  |  |  |  |  |  |  |  |  |  |  |  |  | by PCR of participant |  |  | no | by PCR of participant | | yes |  |  |  | R: 0.61–0.95  IQR: 0.68–0.92 | R: 0.94–0.99  IQR: 0.95–0.98 | R: 0.89–0.98  IQR: 0.91–0.96 |  |

| Wurtz et al., 2021 (*preprint*) [41] | France | cross | 1 | *unclear* | yes | yes | yes | yes | *unclear* | *unclear* | *unclear* | 3 w (impr.) | [+]: body odor (none)  [-]: *unclear* | *unclear* | *unclear* | *unclear* | *unclear* | 12 | 138 | db & r | no | - | 0.08 | 0.92 | 0.96 | 0.96 | overall (one general contingency table per DTE); additional metrics calculated from published results |
| --- | --- | --- | --- | --- | --- | --- | --- | --- | --- | --- | --- | --- | --- | --- | --- | --- | --- | --- | --- | --- | --- | --- | --- | --- | --- | --- | --- |
|  |  |  |  |  |  |  |  |  |  |  |  |  |  |  |  |  | no | by PCR of participant | | yes |  |  |  | - | - | - |  |

| **general** | | | **dogs** | | | **samples** | | | | | **training** | | | | | | **diagnostic test evaluation (DTE)** | | | | | | | **results (diagnostic metrics for DTEs)** | | | | |
| --- | --- | --- | --- | --- | --- | --- | --- | --- | --- | --- | --- | --- | --- | --- | --- | --- | --- | --- | --- | --- | --- | --- | --- | --- | --- | --- | --- | --- |
| **report* [ref]** | **country** | **study design** | **N** | **skull anatomy** | **previous experience in scent work?** | **multiple sample sources/ variation for [+] and [-] samples included (for DTE)?** | **Different stages (symptomatic/ asymptomatic) of COVID-19 samples included**  **(for DTE)?** | **Other diseases, symptoms, or pathogens in [-] samples included (for DTE)?** | **Sample acquisition time for DTE** | **Storage of samples for DTE** | **used scenario** | **duration** | **[+] & [-]**  **sample type (inactivation)** | **approximate sum of positive [+] and negative [-] sample presentations (all dogs)** | | **repeated presentations in at least one dog?** | **used scenario** | **[+] & [-]**  **sample type (inactivation type)** | **approximate sum of positive [+] and negative [-] sample presentations (all dogs)** | | **single blind (b), double blind (db), and/or randomized (r) sample presentations?** | **repeated sample presentations in at least one dog?** | **mocks and “empty” samples included in diagnostic metrics?** | **proportion of positive sample presentations** | **sensitivity (SEN)** | **specificity (SPE)** | **accuracy (ACC)** | **further information about assessment of diagnostic metrics** |
|  |  |  |  |  |  |  |  |  |  |  |  |  |  | **[+]** | **[-]** |  |  | **Mock or “empty” samples included?** | **[+]** | **[-]** | **novelty of samples in DTE guaranteed?** |  |  |  | **range (R) & interquartile range (IQR)** | **range (R) & interquartile range (IQR)** | **range (R) & interquartile range (IQR)** |  |
|  |  |  |  |  |  |  |  |  |  |  |  |  |  | **definition of sample status (reference)** | |  |  |  | **definition of sample status (reference)** | |  |  |  |  |  |  |  |  |

**Supplementary Table 1** Detailed information and performance overview of the evaluated studies *(continued)*

| Vesga et al., 2021 [40] |  | | | | | | | | | | | | | | | | | | | | | | | | | | | |
| --- | --- | --- | --- | --- | --- | --- | --- | --- | --- | --- | --- | --- | --- | --- | --- | --- | --- | --- | --- | --- | --- | --- | --- | --- | --- | --- | --- | --- |
| – DTE 1 | Colombia | case | 6 | normo-cephalic | *unclear* | yes | *unclear* | *controls were saline samples* | seconds | frozen | line-up (outdoors) | 28 d (overall) | [+]: saliva (heat)  [-]: saline | *unclear,* origin. 3 samples | *unclear* | yes | line-up (outdoors) | [+]: saliva and further samples of upper airways (heat)  [-]: saline (none) | approx. 243 | approx. 2957 | partly b  r | yes, included in diagnostic metrics (but addressed with latent class analysis) | yes | approx. 0.08 | 0.89 | 0.97 | 0.97 | overall (one general contingency table per DTE) |
|  |  |  |  |  |  |  |  |  |  |  |  |  |  | by PCR of participant | |  |  | yes | by PCR of participant | | *unclear for [-]* |  |  |  | - | - | - |  |
| – DTE 2 | Colombia | case | 6 | normo-cephalic | *unclear* | yes | *unclear* | *unclear* | seconds–minutes | frozen | line-up (outdoors) | 21 d (overall) | [+] & [-]:  saliva (heat) | *unclear,* origin. 3 samples | *unclear,* origin. 100 samples | yes | line-up (outdoors) | [+]: saliva and further samples of upper airways (heat)  [-]: saliva (heat) | approx. 132 | approx. 5868 | b & r | yes, included in diagnostic metrics (but addressed with latent class analysis) | - | approx. 0.02 | 0.96 | 1.0 | 1.0 |  |
|  |  |  |  |  |  |  |  |  |  |  |  |  |  | by PCR of participant | |  |  | no | by PCR of participant | | no |  |  |  | - | - | - |  |
| – DTE 3 | Colombia | case | 5 | normo-cephalic | *unclear* | yes | yes | yes | seconds (direct body contact) | direct body contact | line-up (direct body contact) | 56 d (overall) | [+] & [-]:  body odor | unclear (origin. 61 particip.) | unclear (origin. 339 particip.) | *unclear* | line-up  (direct body contact) | [+] & [-]:  body odor | 438 | 3747 | b & r | no | - | 0.1 | 0.96 | 0.95 | 0.95 |  |
|  |  |  |  |  |  |  |  |  |  |  |  |  |  | *unclear* | |  |  | no | by PCR or antigen test of participant | | yes |  |  |  | - | - | - |  |
| – DTE 4 | Colombia | cross | 3 | normo-cephalic | *unclear* | yes | yes | yes | seconds (direct body contact) | direct body contact | *no training conducted, DTE 4 took place 75 d after DTE 3* | | | | | | line-up online screening (direct body contact) | [+] & [-]:  body odor | 51 | 1599 | db & r | no | - | 0.03 | 0.69 | 0.94 | 0.94 |  |
|  |  |  |  |  |  |  |  |  |  |  |  |  |  |  |  |  |  | no | by PCR of participant | | yes |  |  |  | - | - | - |  |

| Maia et al., 2021 [56] | Brazil | case | 2 | normo-cephalic/ *unclear* | *unclear* | yes | no | yes | 20 min | cotton pad; cooled; min. 1 w before dog contact | line-up | *unclear* | [+] & [-]:  axillary sweat (none) | *unclear* | yes | line-up | [+] & [-]:  axillary sweat (none) | *unclear,* origin. 44 samples | *unclear,* origin. 56 samples | db & r | no | - | 0.44 | 0.98 | 1.0 | 0.99 | median of all dogs per DTE; additional metrics calculated from published results |
| --- | --- | --- | --- | --- | --- | --- | --- | --- | --- | --- | --- | --- | --- | --- | --- | --- | --- | --- | --- | --- | --- | --- | --- | --- | --- | --- | --- |
|  |  |  |  |  |  |  |  |  |  |  |  |  |  | by PCR of participant |  |  | no | by PCR of participant | | no |  |  |  | R: 0.95–1.0  IQR: 0.95–1.0 | - | R: 0.98–1.0  IQR: 0.98–1.0 |  |

| ten Hagen et al., 2021 [57] |  | | | | | | | | | | | | | | | | | | | | | | | | | | |
| --- | --- | --- | --- | --- | --- | --- | --- | --- | --- | --- | --- | --- | --- | --- | --- | --- | --- | --- | --- | --- | --- | --- | --- | --- | --- | --- | --- |
| – DTE 1 | Germany | case | 8 | normo-cephalic | varied, some COVID-19 experience | yes | *unclear* | yes | seconds | cooled & frozen | DDTS | 3 d (overall) | [+] & [-]:  saliva (BPL) | *unclear* | yes | DDTS | [+] & [-]:  NPS/OPS (BPL) | 189 | 965 | db & r | yes, included in diagnostic metrics | - | 0.16 | 0.73 | 0.95 | 0.93 | median of all dogs per DTE |
|  |  |  |  |  |  |  |  |  |  |  |  |  |  | by PCR of participant |  |  | no | by PCR of participant | | yes |  |  |  | R: 0.6–0.87  IQR: 0.67–0.83 | R: 0.9–1.0  IQR: 0.94–0.98 | R: 0.87–0.96  IQR: 0.89–0.94 |  |
| – DTE 2 | Germany | case | 9 | normo-cephalic | varied, some COVID-19 experience | no | *unclear* | yes | *unclear* | cooled & frozen | DDTS | 3 d (overall) | [+] & [-]:  saliva (BPL) | *unclear* | yes | DDTS | [+] & [-]:  cell culture (BPL) | 110 | 483 | db & r | yes, included in diagnostic metrics | - | 0.19 | 0.57 | 0.93 | 0.87 |  |
|  |  |  |  |  |  |  |  |  |  |  |  |  |  | by PCR of participant |  |  | no | by PCR of participant | | yes |  |  |  | R: 0.46–0.83  IQR: 0.5–0.75 | R: 0.82–0.97  IQR: 0.88–0.95 | R: 0.77–0.91  IQR: 0.8–0.91 |  |
| – DTE 3 | Germany | case | 5 | normo-cephalic | varied, some COVID-19 experience | no | *unclear* | yes | *unclear* | cooled & frozen | DDTS | 3 d (overall) | [+] & [-]:  cell culture (BPL) | *unclear* | yes | DDTS | [+] & [-]:  cell culture (BPL) | 61 | 246 | db & r | yes, included in diagnostic metrics | - | 0.2 | 0.64 | 0.89 | 0.85 |  |
|  |  |  |  |  |  |  |  |  |  |  |  |  |  | by PCR of participant |  |  | no | by PCR of participant | | *unclear* |  |  |  | R: 0.6–1.0  IQR: 0.62–0.96 | R: 0.82–1.0  IQR: 0.84–0.98 | R: 0.82–1.0  IQR: 0.83–0.94 |  |

| Grandjean et al., 2022a [58] | France | case | 7 | normo-cephalic | varied | yes | no | yes | 20 min | gauze; cooled; min. 24 h before dog contact | line-up | 8 w (overall) | [+] & [-]:  axillary sweat (none) | *unclear,* origin. 106 samples | *unclear,* origin. 242 samples | *unclear* | line-up | [+] & [-]:  axillary sweat (none) | 430 | 1080 | db & r | no | - | 0.28 | 0.89 | 0.9 | 0.87 | median of all dogs per DTE; additional metrics calculated from published results |
| --- | --- | --- | --- | --- | --- | --- | --- | --- | --- | --- | --- | --- | --- | --- | --- | --- | --- | --- | --- | --- | --- | --- | --- | --- | --- | --- | --- | --- |
|  |  |  |  |  |  |  |  |  |  |  |  |  |  | by PCR of participant | |  |  | no | by PCR of participant | | yes |  |  |  | R: 0.6–0.94  IQR: 0.87–0.91 | R: 0.78–0.92  IQR: 0.85–0.91 | R: 0.81–0.92  IQR: 0.82–0.91 |  |

| **general** | | | **dogs** | | | **samples** | | | | | **training** | | | | | | **diagnostic test evaluation (DTE)** | | | | | | | **results (diagnostic metrics for DTEs)** | | | | |
| --- | --- | --- | --- | --- | --- | --- | --- | --- | --- | --- | --- | --- | --- | --- | --- | --- | --- | --- | --- | --- | --- | --- | --- | --- | --- | --- | --- | --- |
| **report* [ref]** | **country** | **study design** | **N** | **skull anatomy** | **previous experience in scent work?** | **multiple sample sources/ variation for [+] and [-] samples included (for DTE)?** | **Different stages (symptomatic/ asymptomatic) of COVID-19 samples included**  **(for DTE)?** | **Other diseases, symptoms, or pathogens in [-] samples included (for DTE)?** | **Sample acquisition time for DTE** | **Storage of samples for DTE** | **used scenario** | **duration** | **[+] & [-]**  **sample type (inactivation)** | **approximate sum of positive [+] and negative [-] sample presentations (all dogs)** | | **repeated presentations in at least one dog?** | **used scenario** | **[+] & [-]**  **sample type (inactivation type)** | **approximate sum of positive [+] and negative [-] sample presentations (all dogs)** | | **single blind (b), double blind (db), and/or randomized (r) sample presentations?** | **repeated sample presentations in at least one dog?** | **mocks and “empty” samples included in diagnostic metrics?** | **proportion of positive sample presentations** | **sensitivity (SEN)** | **specificity (SPE)** | **accuracy (ACC)** | **further information about assessment of diagnostic metrics** |
|  |  |  |  |  |  |  |  |  |  |  |  |  |  | **[+]** | **[-]** |  |  | **Mock or “empty” samples included?** | **[+]** | **[-]** | **novelty of samples in DTE guaranteed?** |  |  |  | **range (R) & interquartile range (IQR)** | **range (R) & interquartile range (IQR)** | **range (R) & interquartile range (IQR)** |  |
|  |  |  |  |  |  |  |  |  |  |  |  |  |  | **definition of sample status (reference)** | |  |  |  | **definition of sample status (reference)** | |  |  |  |  |  |  |  |  |

**Supplementary Table 1** Detailed information and performance overview of the evaluated studies *(continued)*

| Grandjean et al., 2022b [59] | France | case | 2 | *unclear* | *unclear* | yes | no | *unclear* | 5 min | surgical swab | *unclear* | line-up | [+] & [-]:  axillary sweat (none) | multiple dogs provided a unique status for each sample (origin. n=45) | multiple dogs provided a unique status for each sample (origin. n=188) | b & r | *unclear* | - | 0.19  (based on sample count, not on sample status definition by dogs) | 0.51 | 1.0 | 0.91 | overall (one general contingency table per DTE); final sample status definition by dogs is unclear: individual decisions of dogs not provided; additional metrics calculated from published results |
| --- | --- | --- | --- | --- | --- | --- | --- | --- | --- | --- | --- | --- | --- | --- | --- | --- | --- | --- | --- | --- | --- | --- | --- |
|  |  |  |  |  |  |  |  |  |  |  |  |  | no | *unclear* | | *unclear* |  |  |  | - | - | - |  |

| Devillier et al., 2022 [60] |  | | | | | | | | | | | | | | | | | | | | | | | | | | |
| --- | --- | --- | --- | --- | --- | --- | --- | --- | --- | --- | --- | --- | --- | --- | --- | --- | --- | --- | --- | --- | --- | --- | --- | --- | --- | --- | --- |
| – DTE 1 | France | case | 7 | normo-cephalic | varied, some COVID-19 experience | no | yes | yes | 10 min | gauze; cooled; min. 72 h before dog contact | line-up | 8 w (overall) | [+] & [-]:  axillary sweat (none) | *unclear* | *unclear* | line-up | [+] & [-]:  axillary sweat (none) | 428 | 458 | b & r | no | - | 0.48 | 0.9 | 0.84 | 0.87 | overall (one general contingency table per DTE) |
|  |  |  |  |  |  |  |  |  |  |  |  |  |  | *unclear* |  |  | no | PCR not used in all individuals ([-] samples) | | yes |  |  |  | - | - | - |  |
| – DTE 2 | France | case | 4 | normo-cephalic | varied, some COVID-19 experience | no | yes | yes | 10 min | surgical mask: cooled; min 72 h before dog contact | line-up | 8 w (overall) | [+] & [-]:  axillary sweat (none) | *unclear* | *unclear* | line-up | [+] & [-]:  surgical masks (none) | 262 (overall) | | b & r | no | - | *unclear* | 0.83 | 0.89 | 0.87 |  |
|  |  |  |  |  |  |  |  |  |  |  |  |  |  | *unclear* |  |  | no | PCR not used in all individuals ([-] samples) | | yes |  |  |  | - | - | - |  |

| Chaber et al., 2022 [61] | Australia | case | 15 | normo-cephalic | varied | yes | *unclear* | *unclear* | 1–20 min | gauze; cooled & frozen; max. usage for 2 w after 1^st^ opening | line-up | *unclear* | [+] & [-]:  axillary sweat (none) | *unclear* | *unclear* | line-up | [+] & [-]:  axillary sweat (none) | 803 | 4457 | db & r | yes, but corrected in calculations | - | 0.15 | 0.95 | 0.97 | - | overall (one general contingency table per DTE); repetitions were corrected for SEN and SPE, therefore no further calculations performed |
| --- | --- | --- | --- | --- | --- | --- | --- | --- | --- | --- | --- | --- | --- | --- | --- | --- | --- | --- | --- | --- | --- | --- | --- | --- | --- | --- | --- |
|  |  |  |  |  |  |  |  |  |  |  |  |  |  | *unclear* |  |  | no | *unclear*, mainly by PCR of participant | | yes |  |  |  | - | - | - |  |

| Guest et al., 2022 [62] |  | | | | | | | | | | | | | | | | | | | | | | | | | | |
| --- | --- | --- | --- | --- | --- | --- | --- | --- | --- | --- | --- | --- | --- | --- | --- | --- | --- | --- | --- | --- | --- | --- | --- | --- | --- | --- | --- |
| – DTE 1 (*appendix*) | UK | case | 3 | normo-cephalic | *unclear* | yes | yes | yes | 12 h (socks)  3 h (masks) | frozen | *unclear* | *unclear* | [+] & [-]:  socks (none)  masks (none) | *unclear* | *unclear* | line-up | [+] & [-]:  socks (none)  masks (none) | *unclear*, origin. 25 samples | *unclear*, origin. 75 samples | partly b  r: *unclear* | *unclear*, metrics assessed for last pass and for blinded part only | - | *unclear* | - | - | - | only range of SEN and SPE provided |
|  |  |  |  |  |  |  |  |  |  |  |  |  |  | by PCR of participant |  |  | no | by PCR of participant | | no |  |  |  | R: 0.76–0.88 | R: 0.9–0.95 | - |  |
| – DTE 2 (*appendix*) | UK | case | 7 (train.)  6 (DTE) | normo-cephalic | *unclear* | yes | yes | yes | 12 h | frozen | line-up | 6–8 w  (overall) | [+] & [-]:  socks (none) | *training samples were used as DTE 🡪* | | line-up | [+] & [-]:  socks (none) | 630 | 1896 | b  r: *unclear* | no | - | 0.25 | 0.78 | 0.94 | 0.9 | median of all dogs per DTE; additional metrics calculated from published results |
|  |  |  |  |  |  |  |  |  |  |  |  |  |  |  |  |  | no | by PCR of participant | | *unclear* |  |  |  | R: 0.75–0.84  IQR: 0.76–0.81 | R: 0.91–0.95  IQR: 0.93–0.94 | R: 0.87–0.93  IQR: 0.89–0.91 |  |
| – DTE 3 | UK | case | 6 | normo-cephalic | *unclear* | yes | yes | yes | 12 h | frozen | *training for DTE 3 = training for DTE 2 and DTE 2 itself* | | | | | line-up | [+] & [-]:  socks (none) | 1129 | 1132 | db & r | no | - | 0.5 | 0.86 | 0.83 | 0.84 |  |
|  |  |  |  |  |  |  |  |  |  |  |  |  |  |  |  |  | no | by PCR of participant | | yes |  |  |  | R: 0.79–0.89  IQR: 0.81–0.89 | R: 0.76–0.89  IQR: 0.8–0.89 | R: 0.81–0.89  IQR: 0.81–0.87 |  |

| **general** | | | **dogs** | | | **samples** | | | | | **training** | | | | | | **diagnostic test evaluation (DTE)** | | | | | | | **results (diagnostic metrics for DTEs)** | | | | |
| --- | --- | --- | --- | --- | --- | --- | --- | --- | --- | --- | --- | --- | --- | --- | --- | --- | --- | --- | --- | --- | --- | --- | --- | --- | --- | --- | --- | --- |
| **report* [ref]** | **country** | **study design** | **N** | **skull anatomy** | **previous experience in scent work?** | **multiple sample sources/ variation for [+] and [-] samples included (for DTE)?** | **Different stages (symptomatic/ asymptomatic) of COVID-19 samples included**  **(for DTE)?** | **Other diseases, symptoms, or pathogens in [-] samples included (for DTE)?** | **Sample acquisition time for DTE** | **Storage of samples for DTE** | **used scenario** | **duration** | **[+] & [-]**  **sample type (inactivation)** | **approximate sum of positive [+] and negative [-] sample presentations (all dogs)** | | **repeated presentations in at least one dog?** | **used scenario** | **[+] & [-]**  **sample type (inactivation type)** | **approximate sum of positive [+] and negative [-] sample presentations (all dogs)** | | **single blind (b), double blind (db), and/or randomized (r) sample presentations?** | **repeated sample presentations in at least one dog?** | **mocks and “empty” samples included in diagnostic metrics?** | **proportion of positive sample presentations** | **sensitivity (SEN)** | **specificity (SPE)** | **accuracy (ACC)** | **further information about assessment of diagnostic metrics** |
|  |  |  |  |  |  |  |  |  |  |  |  |  |  | **[+]** | **[-]** |  |  | **Mock or “empty” samples included?** | **[+]** | **[-]** | **novelty of samples in DTE guaranteed?** |  |  |  | **range (R) & interquartile range (IQR)** | **range (R) & interquartile range (IQR)** | **range (R) & interquartile range (IQR)** |  |
|  |  |  |  |  |  |  |  |  |  |  |  |  |  | **definition of sample status (reference)** | |  |  |  | **definition of sample status (reference)** | |  |  |  |  |  |  |  |  |

**Supplementary Table 1** Detailed information and performance overview of the evaluated studies *(continued)*

| Mancilla-Tapia et al., 2022 [63] |  | | | | | | | | | | | | | | | | | | | | | | | | | |
| --- | --- | --- | --- | --- | --- | --- | --- | --- | --- | --- | --- | --- | --- | --- | --- | --- | --- | --- | --- | --- | --- | --- | --- | --- | --- | --- |
| – DTE 1 | Mexico | case | 4 | normo-cephalic | varied | no | yes | yes | 1 min | gauze and swab; cooled | line-up | 12 w  (overall) | [+] & [-]:  corporal sweat (none) | approx. 2004 | *unclear* | line-up | [+] & [-]:  axillary & corporal sweat (none) | 483 (overall) | b & r (no db as data recorder present in room) | no | no | *unclear* | 0.68 | 0.72 | - | median of all dogs per DTE |
|  |  |  |  |  |  |  |  |  |  |  |  |  |  | by PCR or antigen test of participant |  |  | yes | by PCR of participant, antigen test only in few individuals | yes |  |  |  | R: 0.58–0.8  IQR: 0.59–0.79 | R: 0.64–0.88  IQR: 0.65–0.85 | - |  |
| – DTE 2 | Mexico | case | 3 | normo-cephalic | varied | no | yes | yes | 1 min | swab; cooled | line-up | 12 w  (overall) | [+] & [-]:  saliva (none) | approx. 2004 | *unclear* | line-up | [+]: saliva (none)  [-]: clean swabs (none) | 308 (overall) | b & r (no db as data recorder present in room) | no | no | *unclear* | 0.73 | 0.69 | - |  |
|  |  |  |  |  |  |  |  |  |  |  |  |  |  | by PCR or antigen test of participant |  |  | yes | by PCR of participant, antigen test only in few individuals | *unclear* |  |  |  | R: 0.7–0.78  IQR: 0.7–0.78 | R: 0.53–0.69  IQR: 0.53–0.69 | - |  |

| Maurer et al., 2022 [44] |  | | | | | | | | | | | | | | | | | | | | | | | | | | | |
| --- | --- | --- | --- | --- | --- | --- | --- | --- | --- | --- | --- | --- | --- | --- | --- | --- | --- | --- | --- | --- | --- | --- | --- | --- | --- | --- | --- | --- |
| – DTE 1 | USA | case | 4 (train.)  3 (DTE) | normo-cephalic | no | yes | yes | yes | 15 s | cotton pads, cooled, 72 h before dog contact | line-up | 6 w (overall) | [+] & [-]:  sweat from face/head (none) | approx. 292 | approx. 328 | no | line-up | [+] & [-]:  sweat from face/head (none) | approx. 156 | approx. 624 | db & r | no | - | 0.2 | 0.98 | 0.91 | 0.92 | median of all dogs per DTE; additional metrics calculated from published results |
|  |  |  |  |  |  |  |  |  |  |  |  |  |  | by PCR of participant | |  |  | no | by PCR of participant | | yes |  |  |  | R: 0.96–1.0  IQR: 0.96–1.0 | R: 0.87–0.99  IQR: 0.87–0.99 | R: 0.89–0.99  IQR: 0.89–0.99 |  |
| – DTE 2 | USA | cross (+ addition. samples) | 1 | normo-cephalic | no | yes | yes | yes | 15 s | cotton pads, cooled | training for DTE 2 = training for DTE 1 and DTE 1 itself | | | | | | line-up | [+] & [-]:  sweat from face/head (none) | min. 16 | max. 153 | db & r | no | - | *unclear* | 0.96 | 1.0 | - | overall (one general contingency table per DTE); some samples were added to the cross-sectional design |
|  |  |  |  |  |  |  |  |  |  |  |  |  |  |  |  |  |  | no | by PCR of participant | | yes |  |  |  | - | - | - |  |

| Kantele et al., 2022 [43] |  | | | | | | | | | | | | | | | | | | | | | | | | | | |
| --- | --- | --- | --- | --- | --- | --- | --- | --- | --- | --- | --- | --- | --- | --- | --- | --- | --- | --- | --- | --- | --- | --- | --- | --- | --- | --- | --- |
| – DTE 1 | Finland | case | 9 (train.)  4 (DTE) | normo-cephalic | yes | yes | yes | yes | seconds | gauze, room-temperature, 0­–5 months before dog contact | line-up | *unclear* | [+] & [-]:  sweat from face/throat/neck/wrist (none) | *unclear* | *unclear* | line-up | [+] & [-]:  sweat from face/throat/neck/ wrist (none) | 427 | 882 | db & r | no | - | 0.33 | 0.93 | 0.91 | 0.92 | median of all dogs per DTE; additional metrics calculated from published results |
|  |  |  |  |  |  |  |  |  |  |  |  |  |  | by PCR of participant |  |  | no | by PCR of participant | | yes |  |  |  | R: 0.88–0.94  IQR: 0.89–0.94 | R: 0.9–0.95  IQR: 0.9–0.94 | R: 0.89–0.94  IQR: 0.9–0.935 |  |
| – DTE 2 | Finland | cross (+ addition. samples) | 9 (train.)  4 (DTE) | normo-cephalic | yes | yes | yes | yes | seconds | gauze, room-temperature | training for DTE 2 = training for DTE 1 and DTE 1 itself | | | | | line-up | [+] & [-]:  sweat from face/throat/neck/ wrist (none) | multiple dogs provided a unique status for each sample (origin. n=158) | multiple dogs provided a unique status for each sample (origin. n=300) | db & r | no | - | 0.34  (based on sample count, not on sample status definition by dogs) | 0.97 | 0.99 | 0.98 | overall (one general contingency table per DTE); additional metrics calculated from published results; detections of multiple dogs of the same sample defined sample status and metrics; some samples were added to the cross-sectional design |
|  |  |  |  |  |  |  |  |  |  |  |  |  |  |  |  |  | no | by PCR of participant | | yes |  |  |  | - | - | - |  |

| Grandjean et al., 2022c [42] | France | cross (+ com-parative: Ag test vs. dogs) | 7 | normo-cephalic | yes, COVID-19 experience | yes | yes | yes | 2 min | surgical compresses; frozen | line-up | 1–3 w (overall);  ref to [47] | [+] & [-]:  axillary sweat (none) | *unclear* | *unclear* | line-up | [+] & [-]:  axillary sweat (none) | multiple dogs provided a unique status for each sample (origin. n=109) | multiple dogs provided a unique status for each sample (origin. n=226) | db & r | no | , | 0.33  (based on sample count, not on sample status definition by dogs) | 0.97 | 0.91 | 0.93 | overall (one general contingency table per DTE); detections of multiple dogs of the same sample defined sample status and metrics; additional metrics calculated from published results |
| --- | --- | --- | --- | --- | --- | --- | --- | --- | --- | --- | --- | --- | --- | --- | --- | --- | --- | --- | --- | --- | --- | --- | --- | --- | --- | --- | --- |
|  |  |  |  |  |  |  |  |  |  |  |  |  |  | *unclear* |  |  | no | by NPS-PCR of participant and saliva PCR and antigen test | | yes |  |  |  | - | - | - |  |

**Supplementary Table 1** Detailed information and performance overview of the evaluated studies *(continued)*

| **general** | | | **dogs** | | | **samples** | | | | | **training** | | | | | | **diagnostic test evaluation (DTE)** | | | | | | | **results (diagnostic metrics for DTEs)** | | | | |
| --- | --- | --- | --- | --- | --- | --- | --- | --- | --- | --- | --- | --- | --- | --- | --- | --- | --- | --- | --- | --- | --- | --- | --- | --- | --- | --- | --- | --- |
| **report* [ref]** | **country** | **study design** | **N** | **skull anatomy** | **previous experience in scent work?** | **multiple sample sources/ variation for [+] and [-] samples included (for DTE)?** | **Different stages (symptomatic/ asymptomatic) of COVID-19 samples included**  **(for DTE)?** | **Other diseases, symptoms, or pathogens in [-] samples included (for DTE)?** | **Sample acquisition time for DTE** | **Storage of samples for DTE** | **used scenario** | **duration** | **[+] & [-]**  **sample type (inactivation)** | **approximate sum of positive [+] and negative [-] sample presentations (all dogs)** | | **repeated presentations in at least one dog?** | **used scenario** | **[+] & [-]**  **sample type (inactivation type)** | **approximate sum of positive [+] and negative [-] sample presentations (all dogs)** | | **single blind (b), double blind (db), and/or randomized (r) sample presentations?** | **repeated sample presentations in at least one dog?** | **mocks and “empty” samples included in diagnostic metrics?** | **proportion of positive sample presentations** | **sensitivity (SEN)** | **specificity (SPE)** | **accuracy (ACC)** | **further information about assessment of diagnostic metrics** |
|  |  |  |  |  |  |  |  |  |  |  |  |  |  | **[+]** | **[-]** |  |  | **Mock or “empty” samples included?** | **[+]** | **[-]** | **novelty of samples in DTE guaranteed?** |  |  |  | **range (R) & interquartile range (IQR)** | **range (R) & interquartile range (IQR)** | **range (R) & interquartile range (IQR)** |  |
|  |  |  |  |  |  |  |  |  |  |  |  |  |  | **definition of sample status (reference)** | |  |  |  | **definition of sample status (reference)** | |  |  |  |  |  |  |  |  |

| Twele et al., 2022 [64] |  | | | | | | | | | | | | | | | | | | | | | | | | | | |
| --- | --- | --- | --- | --- | --- | --- | --- | --- | --- | --- | --- | --- | --- | --- | --- | --- | --- | --- | --- | --- | --- | --- | --- | --- | --- | --- | --- |
| – DTE 1 | Germany | case | 8 | normo-cephalic | yes, COVID-19 experience | no | yes | yes | seconds–minutes | frozen | DDTS | 2–4 d  (overall) | [+] & [-]:  saliva, urine, arm crook sweat ([+]: BPL, [-]: with & w/o BPL) | *unclear* | yes | DDTS | [+]: saliva, urine, arm crook sweat acute COVID-19 (BPL)  [-]: saliva long COVID (BPL) | 90 | 454 | db & r | yes | - | 0.17 | 0.87 | 0.96 | 0.94 | median of all dogs per DTE |
|  |  |  |  |  |  |  |  |  |  |  |  |  |  | by PCR of participant |  |  | no | by PCR of participant | not determined | yes |  |  |  | R: 0.76–1.0  IQR: 0.73–1.0 | R: 0.88–1.0  IQR: 0.94–0.995 | R: 0.9–1.0  IQR: 0.91–0.98 |  |
| – DTE 2 | Germany | case | 3 | normo-cephalic | yes, COVID-19 experience | no | yes | *unclear* | seconds–minutes | frozen | DDTS | 2–4 d  (overall) | [+] & [-]:  saliva, urine, arm crook sweat ([+]: BPL, [-]: with & w/o BPL) | *unclear* | yes | DDTS | [+]: saliva long COVID (BPL)  [-]: saliva, urine, arm crook sweat healthy controls (with & w/o BPL) | 14 | 49 | db & r | yes | - | 0.22 | 1.0 | 0.94 | 0.95 |  |
|  |  |  |  |  |  |  |  |  |  |  |  |  |  | by PCR of participant |  |  | no | not determined | by PCR of participant | *unclear* |  |  |  | R: 0.83–1.0  IQR: 0.83–1.0 | R: 0.94–1.0  IQR: 0.94–1.0 | R: 0.91–1.0  IQR: 0.91–1.0 |  |
| – DTE 3 | Germany | case | 3 | normo-cephalic | yes, COVID-19 experience | yes | yes | *unclear* | seconds–minutes | frozen | DDTS | 2–4 d  (overall) | [+] & [-]:  saliva, urine, arm crook sweat ([+]: BPL, [-]: with & w/o BPL) | *unclear* | yes | DDTS | [+]: saliva, urine, arm crook sweat acute COVID-19 (BPL)  [-]: saliva, urine, arm crook sweat healthy controls (with & w/o BPL) | 19 | 106 | db & r | yes | - | 0.15 | 0.86 | 0.89 | 0.87 |  |
|  |  |  |  |  |  |  |  |  |  |  |  |  |  | by PCR of participant |  |  | no | by PCR of participant | | *unclear* |  |  |  | R: 0.75–1.0  IQR: 0.75–1.0 | R: 0.86–0.9  IQR: 0.86–0.9 | R: 0.86–0.92  IQR: 0.86–0.92 |  |

| ten Hagen et al., 2022 [45] |  | | | | | | | | | | | | | | | | | | | | | | | | | | |
| --- | --- | --- | --- | --- | --- | --- | --- | --- | --- | --- | --- | --- | --- | --- | --- | --- | --- | --- | --- | --- | --- | --- | --- | --- | --- | --- | --- |
| – DTE 1 | Germany | cross (+ com-parative: Ag test vs. dogs; + addition. samples) | 3 | normo-cephalic | varied, some COVID-19 experience | yes | yes | yes | seconds | samples provided directly (cotton pad) | DDTS/ line-up | DDTS: 3 d 🡪  line-up: 1–2 w (overall) | [+] & [-]:  saliva, urine, arm crook sweat ([+]: BPL, sweat also w/o BPL; [-]: with & w/o BPL (all sample types)) | *unclear* | yes | line-up | [+] & [-]:  arm crook sweat (none) | multiple dogs provided a unique status for each sample (origin. n=9) | multiple dogs provided a unique status for each sample (origin. n=466) | db & r | no | - | 0.02  (based on sample count, not on sample status definition by dogs) | 0.67 | 1.0 | 0.99 | overall (one general contingency table per DTE); detections of multiple dogs of the same sample defined sample status and metrics; some samples were added to the cross-sectional design |
|  |  |  |  |  |  |  |  |  |  |  |  |  |  | by PCR of participant |  |  | no | by PCR of participant  (in addition antigen and dog testing simultaneously in every participant) | | yes |  |  |  | - | - | - |  |
| – DTE 2 | Germany | cross (+ com-parative: Ag test vs. dogs; + addition. samples) | 7 | normo-cephalic | varied, some COVID-19 experience | yes | yes | yes | seconds | samples provided directly (cotton pad) | DDTS/ line-up | DDTS: 3 d 🡪  line-up: 1–2 w (overall) | [+] & [-]:  saliva, urine, arm crook sweat ([+]: BPL, sweat also w/o BPL; [-]: with & w/o BPL (all sample types)) | *unclear* | yes | line-up | [+] & [-]:  arm crook sweat (none) | multiple dogs provided a unique status for each sample (origin. n=10) | multiple dogs provided a unique status for each sample (origin. n=640) | db & r | no | - | 0.02  (based on sample count, not on sample status definition by dogs) | 0.8 | 1.0 | 1.0 | overall (one general contingency table per DTE); detections of multiple dogs of the same sample defined sample status and metrics; some samples were added to the cross-sectional design |
|  |  |  |  |  |  |  |  |  |  |  |  |  |  | by PCR of participant |  |  | no | by PCR of participant  (in addition antigen and dog testing simultaneously in every participant) | | yes |  |  |  | - | - | - |  |

| **general** | | | **dogs** | | | **samples** | | | | | **training** | | | | | | **diagnostic test evaluation (DTE)** | | | | | | | **results (diagnostic metrics for DTEs)** | | | | |
| --- | --- | --- | --- | --- | --- | --- | --- | --- | --- | --- | --- | --- | --- | --- | --- | --- | --- | --- | --- | --- | --- | --- | --- | --- | --- | --- | --- | --- |
| **report* [ref]** | **country** | **study design** | **N** | **skull anatomy** | **previous experience in scent work?** | **multiple sample sources/ variation for [+] and [-] samples included (for DTE)?** | **Different stages (symptomatic/ asymptomatic) of COVID-19 samples included**  **(for DTE)?** | **Other diseases, symptoms, or pathogens in [-] samples included (for DTE)?** | **Sample acquisition time for DTE** | **Storage of samples for DTE** | **used scenario** | **duration** | **[+] & [-]**  **sample type (inactivation)** | **approximate sum of positive [+] and negative [-] sample presentations (all dogs)** | | **repeated presentations in at least one dog?** | **used scenario** | **[+] & [-]**  **sample type (inactivation type)** | **approximate sum of positive [+] and negative [-] sample presentations (all dogs)** | | **single blind (b), double blind (db), and/or randomized (r) sample presentations?** | **repeated sample presentations in at least one dog?** | **mocks and “empty” samples included in diagnostic metrics?** | **proportion of positive sample presentations** | **sensitivity (SEN)** | **specificity (SPE)** | **accuracy (ACC)** | **further information about assessment of diagnostic metrics** |
|  |  |  |  |  |  |  |  |  |  |  |  |  |  | **[+]** | **[-]** |  |  | **Mock or “empty” samples included?** | **[+]** | **[-]** | **novelty of samples in DTE guaranteed?** |  |  |  | **range (R) & interquartile range (IQR)** | **range (R) & interquartile range (IQR)** | **range (R) & interquartile range (IQR)** |  |
|  |  |  |  |  |  |  |  |  |  |  |  |  |  | **definition of sample status (reference)** | |  |  |  | **definition of sample status (reference)** | |  |  |  |  |  |  |  |  |

**Supplementary Table 1** Detailed information and performance overview of the evaluated studies *(continued)*

| ten Hagen et al., 2022 [45] *(continued)* |  | | | | | | | | | | | | | | | | | | | | | | | | | | |
| --- | --- | --- | --- | --- | --- | --- | --- | --- | --- | --- | --- | --- | --- | --- | --- | --- | --- | --- | --- | --- | --- | --- | --- | --- | --- | --- | --- |
| – DTE 3 | Germany | cross (+ com-parative: Ag test vs. dogs; + addition. samples) | 7 | normo-cephalic | varied, some COVID-19 experience | yes | yes | yes | seconds | samples provided directly (cotton pad) | DDTS/ line-up | DDTS: 3 d 🡪  line-up: 1–2 w (overall) | [+] & [-]:  saliva, urine, arm crook sweat ([+]: BPL, sweat also w/o BPL; [-]: with & w/o BPL (all sample types)) | *unclear* | yes | line-up | [+] & [-]:  arm crook sweat (none) | multiple dogs provided a unique status for each sample (origin. n=11) | multiple dogs provided a unique status for each sample (origin. n=678) | db & r | no | - | 0.02  (based on sample count, not on sample status definition by dogs) | 0.82 | 1.0 | 1.0 | overall (one general contingency table per DTE); detections of multiple dogs of the same sample defined sample status and metrics; some samples were added to the cross-sectional design |
|  |  |  |  |  |  |  |  |  |  |  |  |  |  | by PCR of participant |  |  | no | by PCR of participant  (in addition antigen and dog testing simultaneously in every participant) | | yes |  |  |  | - | - | - |  |
| – DTE 4 | Germany | cross (+ com-parative: Ag test vs. dogs; + addition. samples) | 8 | normo-cephalic | varied, some COVID-19 experience | yes | yes | yes | seconds | samples provided directly (cotton pad) | DDTS/ line-up | DDTS: 3 d 🡪  line-up: 1–2 w (overall) | [+] & [-]:  saliva, urine, arm crook sweat ([+]: BPL, sweat also w/o BPL; [-]: with & w/o BPL (all sample types)) | *unclear* | yes | line-up | [+] & [-]:  arm crook sweat (none) | multiple dogs provided a unique status for each sample (origin. n=8) | multiple dogs provided a unique status for each sample (origin. n=1018) | db & r | no | - | 0.01  (based on sample count, not on sample status definition by dogs) | 1.0 | 1.0 | 1.0 | overall (one general contingency table per DTE); detections of multiple dogs of the same sample defined sample status and metrics; some samples were added to the cross-sectional design |
|  |  |  |  |  |  |  |  |  |  |  |  |  |  | by PCR of participant |  |  | no | by PCR of participant  (in addition antigen and dog testing simultaneously in every participant) | | yes |  |  |  | - | - | - |  |

| Demirbas et al., 2023 [65] |  | | | | | | | | | | | | | | | | | | | | | | | | | | | |
| --- | --- | --- | --- | --- | --- | --- | --- | --- | --- | --- | --- | --- | --- | --- | --- | --- | --- | --- | --- | --- | --- | --- | --- | --- | --- | --- | --- | --- |
| – DTE 1 | Turkey | case | 1 | normo-cephalic | no | no | *unclear* | *unclear* | 4 h | glass vials,  room-temperature, max. 7–10 d before dog contact (at least for samples w/o inactivation) | line-up | 10 w (overall) | [+] & [-]:  masks (none) | origin. 15 samples | *unclear* | *unclear* | line-up | [+] & [-]:  masks (none & heat) | 26 (origin. 29 [+] samples) | 174 | b & r | *unclear* | *unclear* | 0.15 | 1.0 | 0.98 | 0.99 | overall (one general contingency table per DTE); additional metrics calculated from published results. Authors did not use results from heat-inactivated samples for metrics calculation |
|  |  |  |  |  |  |  |  |  |  |  |  |  |  | by PCR of participant | |  |  | yes | by PCR of participant | | *unclear* |  |  |  | - | - | - |  |
| – DTE 2 | Turkey | case | 2 | normo-cephalic | varied | no | *unclear* | *unclear* | *unclear* | 90 s for UV-inactivation | line-up | *unclear, but 10 w probable* | [+] & [-]:  masks (none) | origin. 15 samples from DTE 1 | *unclear* | *unclear* | line-up | [+] & [-]:  masks (UV) | 48 | 1194 | *unclear* | *unclear* | *unclear* | 0.04 | 0.94 | 0.98 | 0.98 | median of all dogs per DTE; additional metrics calculated from published results |
|  |  |  |  |  |  |  |  |  |  |  |  |  |  | by PCR of participant | |  |  | *unclear* | *unclear for UV-inactivated samples* | | *unclear* |  |  |  | R: 0.91–0.96  IQR: 0.91–0.96 | R: 0.97–0.98  IQR: 0.97–0.98 | R: 0.97–0.98  IQR: 0.97–0.98 |  |
| – DTE 3 | Turkey | case | 2 | normo-cephalic | varied | yes | *unclear* | *unclear* | *unclear* | part of samples provided directly, other part from previous storage | training for DTE3 = training for DTE 2 and DTE 2 itself | | | | | | line-up | [+] & [-]:  masks (*unclear if UV in all samples*) | 86 | *344 (however, it is unclear, if “unknown mask” is defined and confirmed as [-] sample)* | *unclear* | *unclear* | - | 0.25 | 1.0 | 1.0 | 1.0 | overall (one general contingency table per DTE); additional metrics calculated from published results |
|  |  |  |  |  |  |  |  |  |  |  |  |  |  |  |  |  |  | no | by PCR of at least in a part of participants | | *unclear* |  |  |  | - | - |  |  |

* Reports sorted by publication date.

Abbreviations: addition.: additional; Ag: antigen; approx.: approximately; BPL: beta propiolactone; case: case-controlled study; cross: cross-sectional (cohort) study; DDTS: Detection Dog Training System; DTE: diagnostic test evaluation; habit.: habituation; impr.: imprinting (of COVID-19 scent); NPS: nasopharyngeal swab; OPS: oropharyngeal swab; origin.: originally; particip.: participants; PCR: polymerase chain reaction; ref: reference; TBS: tracheobronchial secretions; train.: training; UV: ultraviolet radiation; vs.: versus
