# Supplementary material for: Canine olfactory detection of SARS-CoV-2-infected humans—a systematic review
Source: Ann Epidemiol. 2023 Sep;85:68–85. doi: 10.1016/j.annepidem.2023.05.002 (PMC10195768; doi:10.1016/j.annepidem.2023.05.002)
Supplement: Supplementary file 2 — Supplementary material [file mmc2.docx]

| **report*** | **ref** | **No. DTE over-all** | **No. DTE per study** | **DTE design**  see also section “Quality assessment: Canine scent detection work scores based on Johnen et al.” in the main publication | **short description of trainings and corresponding DTEs extracted from reports (see Supplementary Table 1 for more details)**  **Supplementary Table 2** General information and performance overview of the evaluated studies | **training and testing paradigm** | **x = included analysis by QUADAS-2 [23]**  **(x°= highest quality according to review question)**  see also section “Quality assessment: QUADAS-2 for diagnostic accuracy studies” and Table 1 in the main publication | **x = included analysis by the adapted score system [24]**  **(x°= highest quality according to review question)**  see also section “Quality assessment: Canine scent detection work scores based on Johnen et al.” and Table 2 in the main publication | **dogs’ performance per DTE**  see also section “Assessment of quality of outcome measures” and Figure 5A in the main publication and Supplementary Table 1 | | **dogs’ summarized performance per study (median): all DTEs**  see also section “Detection accuracy of dogs” and Figure 5B in the main publication | | **dogs’ summarized performance per study (median): only highest quality DTEs according to QUADAS-2**  Supplementary material    see also section “QUADAS-2 assessment of studies with least risk of bias: Detection performance” and Figure 5C in the main publication | | **dogs’ summarized performance per study (median): only highest quality DTEs according to the adapted score system**    see also section “Assessment of high-quality studies based on Johnen et al.: Detection performance” and Figure 5D in the main publication | |
| --- | --- | --- | --- | --- | --- | --- | --- | --- | --- | --- | --- | --- | --- | --- | --- | --- |
|  |  |  |  |  |  |  |  |  | **SEN** | **SPE** | **SEN** | **SPE** | **SEN** | **SPE** | **SEN** | **SPE** |
| Jendrny et al., 2020 | [46] | 1 | 1 | one DTE only | training with BPL-inactivated saliva & TBS samples (8 dogs) – DTE with BPL-inactivated saliva & TBS samples (8 dogs) | DDTS | x | x | 0.85 | 0.97 | 0.85 | 0.97 |  |  |  |  |
| Grandjean et al., 2020 | [47] | 2 | 1 | one DTE only | training with noninactivated axillary sweat samples (14 dogs) – DTE with noninactivated axillary sweat samples (6 dogs) | line-up | x | x | 0.95 | 0.96 | 0.95 | 0.96 |  |  |  |  |
| Eskandari et al., 2021 | [48] | 3 | 1 | parallel DTE design | training with noninactivated NPS samples (3 dogs) – DTE with noninactivated NPS samples (3 dogs) | scent-wheel | x | x | 0.6 | 0.9 | 0.73 | 0.915 |  |  |  |  |
|  |  | 4 | 2 |  | training with noninactivated clothes & mask samples (3 dogs) – DTE with noninactivated clothes & mask samples (3 dogs) | scent-wheel | x | x | 0.86 | 0.93 |  |  |  |  |  |  |
| Grandjean et al., 2021 | [49] | 5 | 1 | one DTE only | training with noninactivated axillary sweat samples (21 dogs) – DTE with noninactivated axillary sweat samples (21 dogs) | line-up | x | x | 0.93 |  | 0.93 |  |  |  |  |  |
| Essler et al., 2021 | [50] | 6 | 1 | parallel and successive DTE design (intermediate) | training with detergent-inactivated urine samples (9 dogs) – DTE with detergent-inactivated urine samples (9 dogs) | scent-wheel | x |  | 0.71 | 0.99 | 0.68 | 0.98 |  |  |  |  |
|  |  | 7 | 2 |  | training with detergent-inactivated urine samples (8 dogs) – DTE with heat-inactivated pos. & detergent-inactivated neg. urine samples (8 dogs) | scent-wheel | x |  | 0.75 | 0.98 |  |  |  |  |  |  |
|  |  | 8 | 3 |  | training with detergent-inactivated urine samples (8 dogs) – DTE with heat-inactivated urine samples (8 dogs) | scent-wheel | x |  | 0.62 | 0.98 |  |  |  |  |  |  |
|  |  | 9 | 4 |  | training with heat-inactivated urine samples (9 dogs) – DTE with heat-inactivated urine samples (9 dogs) | scent-wheel | x |  | 0.71 | 0.98 |  |  |  |  |  |  |
|  |  | 10 | 5 |  | training with heat-inactivated urine samples (9 dogs) – DTE with heat-inactivated urine samples (9 dogs) | scent-wheel | x |  | 0.68 | 0.99 |  |  |  |  |  |  |
|  |  | 11 | 6 |  | training with heat-inactivated urine samples (8 dogs) – DTE with heat-inactivated urine samples (8 dogs) | scent-wheel | x | x | 0.18 | 0.41 |  |  |  |  |  |  |
|  |  | 12 | 7 |  | training with heat-inactivated urine samples (9 dogs) – DTE with heat-inactivated saliva samples (9 dogs) | scent-wheel | x |  | 0.22 | 1 |  |  |  |  |  |  |
|  |  | 13 | 8 |  | training with heat-inactivated urine samples (9 dogs) – DTE with heat-inactivated saliva samples (9 dogs) | scent-wheel | x |  | 0.11 | 0.94 |  |  |  |  |  |  |
| Hag-Ali et al., 2021 | [39] | 14 | 1 | one DTE only | training with noninactivated axillary sweat samples (4 dogs) – DTE with noninactivated axillary sweat samples (4 dogs) | line-up | x° | x° | 0.83 | 0.99 | 0.83 | 0.99 | 0.83 | 0.99 | 0.83 | 0.99 |
| Mendel et al., 2021 | [51] | 15 | 1 | successive DTE design | training with UV-inactivated mask samples (4 dogs) – DTE with UV-inactivated mask samples (4 dogs). UV-inactivation for pos. mask samples, unclear for neg. mask samples | scent-wheel | x |  | 0.61 | 0.89 | 0.72 | 0.98 |  |  |  |  |
|  |  | 16 | 2 |  | 1^st^ training and 1^st^ DTE were used as training for 2^nd^ DTE (UV-inactivated pos. mask samples & neg. unused mask samples with unclear inactivation status; 4 dogs) | scent-wheel | x |  | 0.91 | 0.98 |  |  |  |  |  |  |
|  |  | 17 | 3 |  | 1^st^ & 2^nd^ training & DTE were used as training for 3^rd^ DTE (UV-inactivated pos. mask samples & neg. mask samples from healthy participants as well as unused mask samples with unclear inactivation status; 4 dogs) | scent-wheel | x | x | 0.72 | 1 |  |  |  |  |  |  |
| Angeletti et al., 2021 | [52] | 18 | 1 | one DTE only | training with noninactivated axillary sweat samples (3 dogs) – DTE with noninactivated axillary sweat samples (3 dogs) | line-up | x | x | 0.87 |  | 0.87 |  |  |  |  |  |
| Sarkis et al., 2022 | [53] | 19 | 1 | one DTE only | training with noninactivated axillary sweat samples (2 dogs) – DTE with noninactivated axillary sweat samples (2 dogs) | line-up | x | x | 1 | 0.94 | 1 | 0.94 |  |  |  |  |
| Vlachová et al., 2021 (*pre*) | [54] | 20 | 1 | one DTE only | training with noninactivated torso sweat & breath samples (4 dogs) – DTE with noninactivated torso sweat & breath samples (2 dogs) | line-up | x | x | 0.95 | 0.94 | 0.95 | 0.94 |  |  |  |  |
| Jendrny et al., 2021 | [55] | 21 | 1 | parallel DTE design | training with BPL-inactivated saliva samples (10 dogs) – DTE with noninactivated saliva samples (10 dogs) | DDTS | x | x° | 0.84 | 0.95 | 0.84 | 0.96 |  |  | 0.84 | 0.96 |
|  |  | 22 | 2 |  | training with BPL-inactivated saliva samples (10 dogs) – DTE with noninactivated saliva, arm crook sweat, and urine samples (10 dogs) | DDTS | x | x° | 0.53 | 0.96 |  |  |  |  |  |  |
|  |  | 23 | 3 |  | training with BPL-inactivated saliva samples (9 dogs) – DTE with noninactivated arm crook sweat samples (9 dogs) | DDTS | x | x° | 0.91 | 0.94 |  |  |  |  |  |  |
|  |  | 24 | 4 |  | training with BPL-inactivated saliva samples (10 dogs) – DTE with noninactivated urine samples (10 dogs) | DDTS | x | x° | 0.96 | 0.98 |  |  |  |  |  |  |
|  |  | 25 | 5 |  | training with BPL-inactivated saliva samples (10 dogs) – DTE with noninactivated saliva samples (10 dogs) | DDTS | x | x° | 0.82 | 0.96 |  |  |  |  |  |  |
| Wurtz et al., 2021 (*pre*) | [41] | 26 | 1 | one DTE only | training with body odor (1 dog) – DTE procedure unclear (1 dog) | unclear | x | x | 0.92 | 0.96 | 0.92 | 0.96 |  |  |  |  |
| Vesga et al., 2021 | [40] | 27 | 1 | successive DTE design | training with heat-inactivated pos. saliva samples & noninactivated saline samples as neg. controls (6 dogs) – DTE with heat-inactivated pos. saliva as well as upper airways samples & noninactivated saline samples as neg. controls (6 dogs) | line-up | x |  | 0.89 | 0.97 | 0.925 | 0.96 |  |  |  |  |
|  |  | 28 | 2 |  | training with heat-inactivated saliva samples (6 dogs) – DTE with heat-inactivated pos. saliva as well as upper airways samples & heat-inactivated saliva samples as neg. controls (6 dogs) | line-up | x |  | 0.96 | 1 |  |  |  |  |  |  |
|  |  | 29 | 3 |  | training with body odor, direct human contact (5 dogs) – DTE with body odor, direct human contact (5 dogs) | line-up | x | x | 0.96 | 0.95 |  |  |  |  |  |  |
|  |  | 30 | 4 |  | long training gap since 3^rd^ DTE – DTE with body odor, direct human contact (3 dogs) | line-up | x |  | 0.69 | 0.94 |  |  |  |  |  |  |
| Maia et al., 2021 | [56] | 31 | 1 | one DTE only | training with noninactivated axillary sweat samples (2 dogs) – DTE with noninactivated axillary sweat samples (2 dogs) | line-up | x | x | 0.98 | 1 | 0.98 | 1 |  |  |  |  |
| ten Hagen et al., 2021 | [57] | 32 | 1 | parallel DTE design | training with BPL-inactivated saliva samples (8 dogs) – DTE with BPL-inactivated nasopharyngeal & oropharyngeal swabs (8 dogs) | DDTS | x | x | 0.73 | 0.95 | 0.64 | 0.93 |  |  |  |  |
|  |  | 33 | 2 |  | training with BPL-inactivated saliva samples (9 dogs) – DTE with BPL-inactivated cell culture samples (9 dogs) | DDTS | x |  | 0.57 | 0.93 |  |  |  |  |  |  |
|  |  | 34 | 3 |  | training with BPL-inactivated cell culture samples (5 dogs) – DTE with BPL-inactivated cell culture samples (5 dogs) | DDTS | x |  | 0.64 | 0.89 |  |  |  |  |  |  |
| Grandjean et al., 2022a | [58] | 35 | 1 | one DTE only | training with noninactivated axillary sweat samples (7 dogs) – DTE with noninactivated axillary sweat samples (7 dogs) | line-up | x | x | 0.89 | 0.9 | 0.89 | 0.9 |  |  |  |  |
| Grandjean et al., 2022b | [59] | 36 | 1 | one DTE only | training procedure unclear (2 dogs) – DTE with noninactivated axillary sweat samples (2 dogs) | line-up | x | x | 0.51 | 1 | 0.51 | 1 |  |  |  |  |
| Devillier et al., 2022 | [60] | 37 | 1 | parallel DTE design | training with noninactivated axillary sweat samples (7 dogs) – DTE with noninactivated axillary sweat samples (7 dogs) | line-up | x |  | 0.9 | 0.84 | 0.865 | 0.865 |  |  |  |  |
|  |  | 38 | 2 |  | training with noninactivated axillary sweat samples (4 dogs) – DTE with noninactivated surgical mask samples (4 dogs) | line-up | x | x | 0.83 | 0.89 |  |  |  |  |  |  |
| Chaber et al., 2022 | [61] | 39 | 1 | one DTE only | training with noninactivated axillary sweat samples (15 dogs) – DTE with noninactivated axillary sweat samples (15 dogs) | line-up | x | x | 0.95 | 0.97 | 0.95 | 0.97 |  |  |  |  |
| Guest et al., 2022 | [62] | 40** | 1 | successive DTE design | training with noninactivated sock & mask samples (3 dogs) – DTE with noninactivated sock & mask samples (3 dogs); only range of SEN and SPE was provided | line-up | x |  | 0.76–0.88 | 0.9–0.95 |  |  |  |  |  |  |
|  |  | 41** | 2 |  | training with noninactivated sock samples (7 dogs) – DTE with noninactivated sock samples (6 dogs) (training performance was already part of DTE assessment) | line-up | x |  | 0.78 | 0.94 | 0.82 | 0.885 |  |  |  |  |
|  |  | 42 | 3 |  | 2^nd^ training & 2^nd^ DTE were used as training for 3^rd^ DTE (noninactivated sock samples; 6 dogs) | line-up | x | x° | 0.86 | 0.83 |  |  |  |  | 0.86 | 0.83  1 |

Supplementary material

**Supplementary Table 2** General information and performance overview of the evaluated studies *(continued)*

**Supplementary Table 2** General information and performance overview of the evaluated studies

| **report*** | **ref** | **No. DTE over-all** | **No. DTE per study** | **DTE design**  see also section “Quality assessment: Canine scent detection work scores based on Johnen et al.” in the main publication | **short description of trainings and corresponding DTEs extracted from reports (see Supplementary Table 1 for more details)** | **training and testing paradigm** | **x = included analysis by QUADAS-2 [23]**  **(x°= highest quality according to review question)**  see also section “Quality assessment: QUADAS-2 for diagnostic accuracy studies” and Table 1 in the main publication | **x = included analysis by the adapted score system [24]**  **(x°= highest quality according to review question)**  see also section “Quality assessment: Canine scent detection work scores based on Johnen et al.” and Table 2 in the main publication | **dogs’ performance per DTE**  see also section “Assessment of quality of outcome measures” and Figure 5A in the main publication and Supplementary Table 1 | | **dogs’ combined performance per study (median): all DTEs**  see also section “Detection accuracy of dogs” and Figure 5B in the main publication | | **dogs’ combined performance per study (median): only highest quality DTEs according to QUADAS-2**    see also section “QUADAS-2 assessment of studies with least risk of bias: Detection performance” and Figure 5C in the main publication | | **dogs’ combined performance per study (median): only highest quality DTEs according to the adapted score system**    see also section “Assessment of high-quality studies based on Johnen et al.: Detection performance” and Figure 5D in the main publication | |
| --- | --- | --- | --- | --- | --- | --- | --- | --- | --- | --- | --- | --- | --- | --- | --- | --- |
|  |  |  |  |  |  |  |  |  | **SEN** | **SPE** | **SEN** | **SPE** | **SEN** | **SPE** | **SEN** | **SPE** |
| Mancilla-Tapia et al., 2022 | [63] | 43 | 1 | parallel DTE design | training with noninactivated corporal sweat samples (4 dogs) – DTE with noninactivated axillary & corporal sweat samples (4 dogs) | line-up | x | x | 0.68 | 0.72 | 0.705 | 0.705 |  |  |  |  |
|  |  | 44 | 2 |  | training with noninactivated saliva samples (3 dogs) – DTE with noninactivated pos. saliva samples & noninactivated clean swabs as neg. control (3 dogs) | line-up | x |  | 0.73 | 0.69 |  |  |  |  |  |  |
| Maurer et al., 2022 | [44] | 45 | 1 | successive DTE design | training with noninactivated head/face sweat samples (4 dogs) – DTE with noninactivated head/face sweat samples (3 dogs) | line-up | x | x | 0.98 | 0.91 | 0.97 | 0.96 |  |  |  |  |
|  |  | 46 | 2 |  | 1^st^ training & 1^st^ DTE were used as training for 2^nd^ DTE (noninactivated head/face sweat samples; 1 dog) | line-up | x |  | 0.96 | 1 |  |  |  |  |  |  |
| Kantele et al., 2022 | [43] | 47 | 1 | successive DTE design | training with noninactivated sweat samples from face/throat/neck/wrist (9 dogs) – DTE with noninactivated sweat samples from face/throat/neck/wrist (4 dogs) | line-up | x |  | 0.93 | 0.91 | 0.95 | 0.95 |  |  |  |  |
|  |  | 48 | 2 |  | 1^st^ training & 1^st^ DTE were used as training for 2^nd^ DTE (noninactivated sweat samples from face/throat/neck/wrist; 4 dogs) | line-up | x° | x° | 0.97 | 0.99 |  |  | 0.97 | 0.99 | 0.97 | 0.99 |
| Grandjean et al., 2022c | [42] | 49 | 1 | one DTE only | training with noninactivated axillary sweat samples (7 dogs) – DTE with noninactivated axillary sweat samples (7 dogs) | line-up | x° | x° | 0.97 | 0.91 | 0.97 | 0.91 | 0.97 | 0.91 | 0.97 | 0.91 |
| Twele et al., 2022 | [64] | 50 | 1 | parallel DTE design | training with BPL-inactivated pos. + neg. & noninactivated neg. saliva, arm crook sweat & urine samples (8 dogs) – DTE with BPL-inactivated acute pos. samples (saliva/arm crook sweat/urine) vs. BPL-inactivated long COVID saliva samples (neg. controls) (8 dogs) | DDTS | x | x | 0.87 | 0.96 | 0.87 | 0.94 |  |  |  |  |
|  |  | 51 | 2 |  | training with BPL-inactivated pos. + neg. & noninactivated neg. saliva, arm crook sweat & urine samples (3 dogs) – DTE with BPL-inactivated long COVID saliva samples vs. BPL-inactivated & noninactivated neg. saliva/arm crook sweat/urine control samples (3 dogs) | DDTS | x |  | 1 | 0.94 |  |  |  |  |  |  |
|  |  | 52 | 3 |  | training with BPL-inactivated pos. + neg. & noninactivated neg. saliva, arm crook sweat & urine samples (3 dogs) – DTE with BPL-inactivated acute pos. samples (saliva/arm crook sweat/urine) vs. BPL-inactivated & noninactivated neg. saliva/arm crook sweat/urine controls (3 dogs) | DDTS | x |  | 0.86 | 0.89 |  |  |  |  |  |  |
| ten Hagen et al., 2022 | [45] | 53 | 1 | parallel DTE design | training with BPL-inactivated pos. saliva/arm crook sweat/urine as well as noninactivated pos. arm crook sweat samples & neg. BPL-inactivated and noninactivated saliva/arm crook sweat/urine samples (3 dogs) – DTE with noninactivated arm crook sweat samples (3 dogs) | DDTS/line-up | x° |  | 0.67 | 1 | 0.81 | 1 | 0.81 | 1 |  |  |
|  |  | 54 | 2 |  | training with BPL-inactivated pos. saliva/arm crook sweat/urine as well as noninactivated pos. arm crook sweat samples & neg. BPL-inactivated and noninactivated saliva/arm crook sweat/urine samples (7 dogs) – DTE with noninactivated arm crook sweat samples (7 dogs) | DDTS/line-up | x° | x° | 0.8 | 1 |  |  |  |  | 0.82 | 1 |
|  |  | 55 | 3 |  | training with BPL-inactivated pos. saliva/arm crook sweat/urine as well as noninactivated pos. arm crook sweat samples & neg. BPL-inactivated and noninactivated saliva/arm crook sweat/urine samples (7 dogs) – DTE with noninactivated arm crook sweat samples (7 dogs) | DDTS/line-up | x° | x° | 0.82 | 1 |  |  |  |  |  |  |
|  |  | 56 | 4 |  | training with BPL-inactivated pos. saliva/arm crook sweat/urine as well as noninactivated pos. arm crook sweat samples & neg. BPL-inactivated and noninactivated saliva/arm crook sweat/urine samples (8 dogs) – DTE with noninactivated arm crook sweat samples (8 dogs) | DDTS/line-up | x° | x° | 1 | 1 |  |  |  |  |  |  |
| Demirbas et al., 2023 | [65] | 57 | 1 | successive DTE design | training with noninactivated mask samples (1 dog) – DTE with heat-inactivated & noninactivated mask samples (1 dog) | line-up | x |  | 1 | 0.98 | 1 | 0.98 |  |  |  |  |
|  |  | 58 | 2 |  | training with noninactivated mask samples (2 dogs) – DTE with UV-inactivated mask samples (2 dogs) | line-up | x |  | 0.94 | 0.98 |  |  |  |  |  |  |
|  |  | 59 | 3 |  | 2^nd^ training and 2^nd^ DTE were used as training for 3^rd^ DTE (UV-inactivated mask samples; unclear if all samples UV-inactivated) (2 dogs) | line-up | x | x | 1 | 1 |  |  |  |  |  |  |

* Reports sorted by publication date. ** DTEs described in appendix.

Abbreviations: BPL: beta propiolactone; DDTS: Detection Dog Training System; DTE: diagnostic test evaluation; neg: negative; NPS: nasopharyngeal secretion; pos.: positive; pre: preprint; ref: reference; SEN: sensitivity; SPE: specificity; TBS: tracheobronchial secretion; UV: ultraviolet radiation;

2

vs.: versus
