# Supplementary material for: Canine olfactory detection of SARS-CoV-2-infected humans—a systematic review
Source: Ann Epidemiol. 2023 Sep;85:68–85. doi: 10.1016/j.annepidem.2023.05.002 (PMC10195768; doi:10.1016/j.annepidem.2023.05.002)
Supplement: Supplementary file 3 — Supplementary material [file mmc3.docx]

**Supplementary Table 3** Publications and reasons for exclusion in stage 2 of the study search process of the systematic review

| **report*** | **reason for exclusion** |
| --- | --- |
| Emmanuel André, Olivier Denis, Herman Goossens, Marie Pierre Hayette, Yves Lafort, Barbara Legiest, et al. L'utilisation De Chiens Renifleurs Pour La Détection Du Sars-Cov-2. Sciensano [Preprint] (2021). Available at: https://covid-19.sciensano.be/sites/default/files/Covid19/20210412_ Advice%20RAG_Use%20of%20search% 20dogs%20for%20detecting%20SARS-CoV-2_FR.pdf [Last access on 27 July 2022] | preprint information was sparse and it is not clear if inclusion criterion iii) (reference standard) was conducted appropriately in all positive and negative samples |
| Anne-Lise Chaber, Susan Hazel, Brett Matthews, Alexander Withers, Guillaume Alvergnat, Dominique Grandjean, Charles Caraguel. Evaluation Of Canine Detection Of Covid-19 Infected Individuals Under Controlled Settings. Authorea [Preprint] (2021). Available at: https://www.authorea.com/users/412712/articles/521301-evaluation-of-canine-detection-of-covid-19-infected-individuals-under-controlled-settings; doi: 10.22541/au.162055614.42295814/v1 [Last access on 27 July 2022] | obsolete preprint, published in a peer reviewed journal [61] |
| Dominique Grandjean, Caroline Elie, Capucine Gallet, Clotilde Julien, Vinciane Roger, Loïc Desquilbet, et al. Diagnostic Accuracy Of Non-Invasive Detection Of Sars-CoV-2 Infection By Canine Olfaction. medRxiv [Preprint] (2022). Available at: https://www.medrxiv.org/content/10.1101/2022.03.07.22271219v1; doi: 10.1101/2022.03.07.22271219 [Last access on 27 July 2022] | obsolete preprint, published in a peer reviewed journal [42] |
| Dominique Grandjean, Capucine Gallet, Clotilde Lecoq-Julien, Riad Sarkis, QuentinMuzzin, Vinciane Roger, et al. Sars-CoV-2 Virus Infected Patient Identification Through Canine Olfactive Detection On Axillary Sweat Samples.  medRxiv [Preprint] (2021). Available at: https://www.medrxiv.org/content/10.1101/2021.06.10.21257898v1; doi: 10.1101/2021.06.10.21257898 [Last access on 27 July 2022] | obsolete preprint, published in a peer reviewed journal [58] |
| Dominique Grandjean, Dana Humaid Al Marzooqi, Clothilde Lecoq-Julien, QuentinMuzzin, Hamad Katir Al Hammadi, et al. Use Of Canine Olfactory Detection For COVID-19 Testing Study On U.A.E. Trained Detection Dog Sensitivity. bioRxiv [Preprint] (2021). Available at: https://www.biorxiv.org/content/10.1101/2021.01.20.427105v1; doi: 10.1101/2021.01.20.427105 [Last access on 27 July 2022] | obsolete preprint, published in a peer reviewed journal [49] |
| Dominique Grandjean, Riad Sarkis, Jean Pierre Tourtier, Clothilde JulienLecocq, Aymeric Benard, Vinciane Roger, et al. Detection dogs as a help in the detection of COVID-19 Can the dog alert on COVID-19 positive persons by sniffing axillary sweat samples ? Proof-of-concept study. bioRxiv [Preprint] (2020). Available at: https://www.biorxiv.org/content/10.1101/2020.06.03.132134v1; doi: 10.1101/2020.06.03.132134 [Last access on 27 July 2022] | obsolete preprint, published in a peer reviewed journal [47] |
| Dominique Grandjean, Dorsaf Slama, Capucine Gallet, Clothilde Julien, Emilie Seyrat, Marc Blondot, et al. Screening for SARS-CoV-2 persistence in Long COVID patients using sniffer dogs and scents from axillary sweats samples. medRxiv [Preprint] (2022). Available at: https://www.medrxiv.org/content/10.1101/2022.01.11.21268036v1; doi: 10.1101/2022.01.11.21268036 [Last access on 27 July 2022] | obsolete preprint, published in a peer reviewed journal [59] |
| Claire Guest, Sarah Y Dewhirst, David J Allen, Sophie Aziz, Oliver Baerenbold, John Bradley, et al. Using trained dogs and organic semi-conducting sensors to identify asymptomatic and mild SARS-CoV-2 infections. Lshtm [Preprint] (2021). Available at: https://www.lshtm.ac.uk/media/49791 [Last access on 27 July 27 2022] | obsolete preprint, published in a peer reviewed journal [62] |
| Paula Jendrny, Friederike Twele, Sebastian Meller, Claudia Schulz, Maren von Köckritz-Blickwede, Ab Osterhaus, et al. Scent dog identification of SARS-CoV-2 infections, similar across different body fluids. bioRxiv [Preprint] (2021). Available at: https://www.biorxiv.org/content/10.1101/2021.03.05.434038v1; doi: 10.1101/2021.03.05.434038 [Last access on 27 July 2022] | obsolete preprint, published in a peer reviewed journal [55] |
| Yasemin Salgirli, Gorkem Kismali, Begum Saral, Baris Sareyyüpoğlu, Arif Dogan Habiloğlu, Hakan Öztürk, et al. Development of a Safety Protocol for Training and Using SARS-CoV-2 Detection Dogs. J Vet Behav [Preprint] (2022). Available at: https://papers.ssrn.com/sol3/papers.cfm?abstract_id=4075240# [Last access on 6 July 2022] | obsolete preprint, published in a peer reviewed journal [65] |
| Nele Alexandra ten Hagen, Friederike Twele, Sebastian Meller, Lisa Wijnen, Claudia Schulz, Clara Schoneberg, et al. Canine real-time detection of SARS-CoV-2 infections in the context of a mass screening event. BMJ Glob Heal [in revision]. | obsolete manuscript, published in a peer reviewed journal [45] |
| Omar Vesga, Maria Agudelo, Andrés F. Valencia-Jaramillo, Alejandro Mira-Montoya, Felipe Ossa-Ospina, Esteban Ocampo, et al. Highly sensitive scent-detection of COVID-19 patients in vivo by trained dogs. medRxiv [Preprint] (2021). Available at: https://www.medrxiv.org/content/10.1101/2021.05.30.21257913v1; doi: 10.1101/2021.05.30.21257913 [Last access on 27 July 2022] | obsolete preprint, published in a peer reviewed journal [40] |
| Omar Vesga, Andres F. Valencia, Alejandro Mira, Felipe Ossa, Esteban Ocampo, Maria Agudelo, et al. Dog Savior: Immediate Scent-Detection of SARS-COV-2 by Trained Dogs. bioRxiv [Preprint] (2020). Available at: https://www.biorxiv.org/content/10.1101/2020.06.17.158105v1; doi: 10.1101/2020.06.17.158105 [Last access on 27 July 2022] | obsolete preprint, published in a peer reviewed journal [40] |

**Supplementary Table 3** Publications and reasons for exclusion in stage 2 of the study search process of the systematic review *(continued)*

| **report*** | **reason for exclusion** |
| --- | --- |
| Nathalie Wurtz, Alexandre Lacoste, Bernard La Scola. Real-time detection of Covid-19 positive persons using sniffer dog. Infectious Diseases (2022) 54:384-386. doi: 10.1080/23744235.2022.2033311 | based on the title but due to the lack of an abstract the study entered the second stage. Full text screening not possible since article was not available, also on request. This study was published as a Letter to the Editor and was not considered peer-reviewed. It corresponds to the analyzed preprint of Wurtz et al. [41] and was considered equivalent. |

* Reports sorted alphabetically by the first author’s surname.
